# Macrocyclic Copper(II) Electrocatalysts for Water Oxidation: Catalytic Mechanism and Activity of Pyridine-Embedded Complexes

**DOI:** 10.1021/acs.jpca.6c01912

**Published:** 2026-06-23

**Authors:** João Pedro C. S. Neves, Roberto Rivelino, Tiago Vinicius Alves, Vitor H. Menezes da Silva

**Affiliations:** † Departamento de Físico-Química, 495510 Instituto de Química, Universidade Federal da Bahia, Rua Barão de Jeremoabo, Salvador, Bahia 40170-115, Brazil; ‡ Instituto de Física, 28111 Universidade Federal da Bahia, Salvador, Bahia 40210-340, Brazil; § Departamento de Química, Centro de Ciências Exatas, 37894 Universidade Estadual de Londrina, Rodovia Celso Garcia Cid, PR 445 Km 380, Londrina 86050-482, Paraná, Brasil

## Abstract

Electrochemical water oxidation (WO) plays a central role in hydrogen production strategies for the energy transition, as it constitutes the main kinetic bottleneck of the overall process. Herein, a computational study was carried out to investigate the mechanism and activity of [Cu­(Me_3_Pyclen)]^2+^ as an electrocatalyst toward WO, involving the pyridine-embedded macrocylic ligand Me_3_Pyclen (Me_3_Pyclen = 4,7,10-trimethyl-1,4,7,10-tetraaza-2,6-pyridinophane). Several pyridine-embedded Cu complexes at different electronic states have been computationally determined for the electrooxidation steps, revealing an intricate mechanism for the catalyst activation. Subsequently, the TOF-determining step of the catalytic cycle was estimated to be the O–O coupling via two viable reaction pathways: through single- or double-water coordination modes. In the first pathway, a buffer-mediated water nucleophilic attack coupled with single-electron transfer (SET-WNA) is involved, whereas the second proceeds through an intramolecular oxygen coupling (IOC) mechanism. Additionally, outer-sphere electron transfer steps might play an important role in generating reactive species that promote the O–O coupling. Overall, through a detailed theoretical screening of electrocatalytic reaction steps, we were able to compare the distinct mechanistic features of these electrocatalysts based on their electronic and steric features, possibly opening new possibilities for the rational design of ligands for WO catalyzed by Cu complexes.

## Graphical abstract

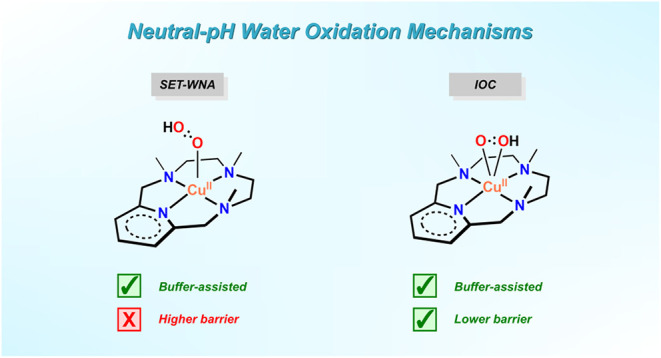

## Introduction

The global pursuit of sustainability is fundamentally tied to the transformation of our energy infrastructure.^1^ In this sense, the energy transition from the use of fossil fuels to clean and renewable sources remains a major scientific and technological challenge, thus reducing carbon emissions and mitigating climate change. To this end, significant advances have been achieved in materials and catalysts.^2,3^ In this scenario, molecular hydrogen (H_2_) has emerged as a compelling energy carrier due to its strong chemical bond; furthermore, the H_2_ combustion releases water as the main product.^4^ One of the many ways to produce H_2_ involves the electrolysis of water (eq 1),^5^ for which the anodic half-reaction, water oxidation (WO), constitutes the primary kinetic bottleneck.^6^ This drawback arises from the transfer of four protons and four electrons from two water molecules, along with the O–O coupling, as depicted in the equation in Scheme 1. Thereby, to enhance the sustainable H_2_ production from water, the development of biomimetic molecular WO catalysis based on earth-abundant 3*d* transition-metal complexes has become a subject of extensive investigation in recent years.^7,8^

1 $$2H_{2}O\to O_{2}+2H_{2},E_{\mathrm{SHE}}^{{}^{\circ}}=-1.23\;V$$

**Scheme 1:**
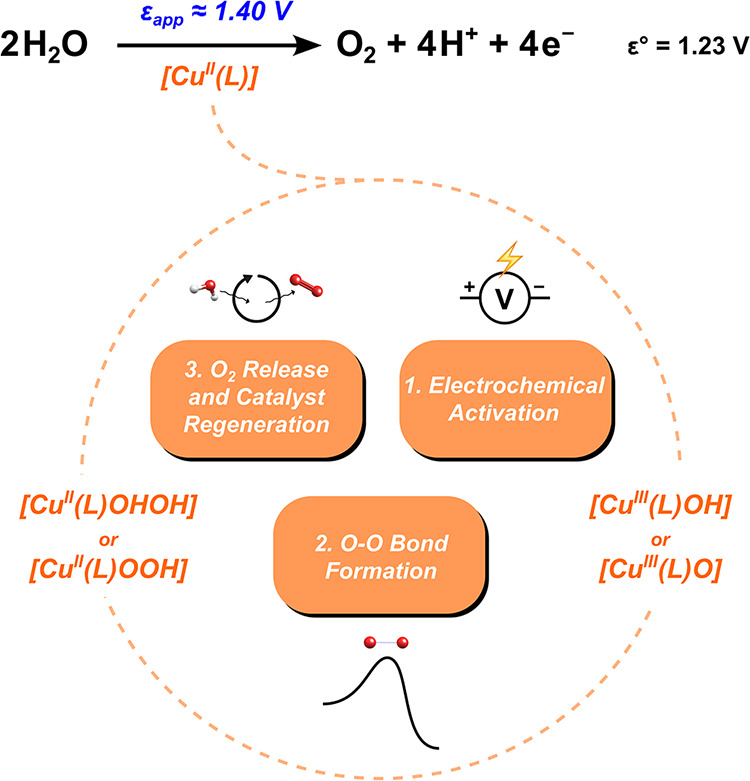
WO Catalytic Cycle By Mononuclear Cu^II^ Complexes Generalized in Three Main Parts; for the Sake of Simplicity, the Ligand L was Considered as Redox innocent

Among such catalytic systems, mononuclear copper­(II) complexes stand out as some of the most explored catalysts from both experimental^9–11^ and theoretical^12–17^ perspectives. The advantage of copper complexes resides in their considerable activity at low overpotentials and the possibility of high catalytic activity for WO under neutral pH conditions. In this direction, the electronic and steric features of Cu complex ligands (L) are crucial, providing consequences for the overall mechanistic sequence of the catalytic cycle, as shown in Scheme 1. Specifically in the first step, after water coordination to the Cu ion center, a series of single-electron transfers (SET) and/or proton-coupled electron transfers (PCET) are necessary for the electrochemical activation of the catalyst, in which the electron transfers can be either metal- or ligand-centered, thus generating the active oxidized intermediates that subsequently drive the O–O bond coupling. In most cases, the process of O–O bond formation accounts for the energy barrier of the overall half-reaction, i.e., it is the rate-determining step of WO.^18^ The final steps usually consist of strongly exergonic electrochemical events leading to O_2_ generation, so that the protons and electrons are delivered in combination for the formation of H_2_ at the cathode.

The first-order O–O bond formation can occur through different mechanisms depending on the nature of the metal center or the ligand environment. Early catalysts based on noble metals or even first-half 3*d* transition metals, for example, usually follows a direct water nucleophilic attack (WNA) on the oxo group of the complex. These species may also undergo a second-order interaction of two metals (I2M) leading to the O–O coupling.^19^ For late 3*d* transition metal catalysts such as copper, the typical mechanism proceeds through a SET-coupled water nucleophilic attack (SET-WNA) on a coordinated oxygen of the complex, or by an intramolecular oxygen coupling (IOC), as depicted in Scheme 2.^6,18,20^ Under neutral pH conditions, the SET-WNA also requires the assistance of proton-withdrawing buffer species from the aqueous medium, which may or may not take place concertedly with respect to the O–O bond stretching and the SET to the oxidized metal/ligand framework. On the other hand, in alkaline media, the water nucleophilic attack (WNA) process occurs via hydroxide nucleophilic attack, even though second-sphere interactions are potentially relevant to enable the O–O bond formation.^13,15^ Regarding the IOC, which is often associated with lower energy barriers, a “scissor” vibrational mode, i.e., the ∠O–Cu–O bending, leads to the O–O coupling.^21^ The IOC pathway, however, has been evidenced in selected cases for mononuclear Cu­(II) complexes,^22^ since the steric effects of the ligand are important to enable the presence of two vicinal water molecules in their coordination environment, thus paving a new way for designing robust molecular WO electrocatalysts.

**Scheme 2:**
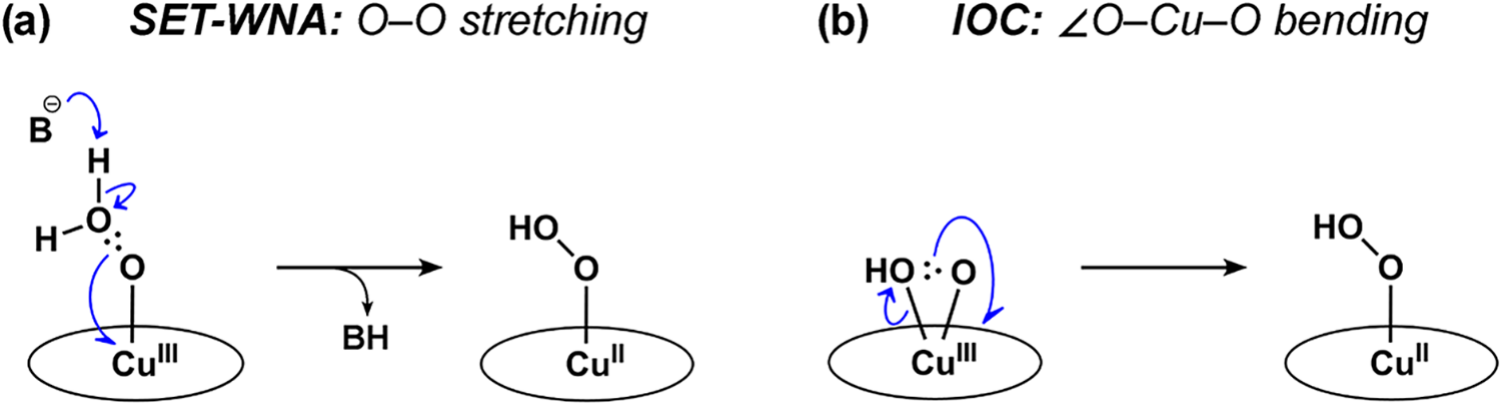
Simplified Lewis Structures of Two O–O Bond Formation Mechanisms from an Electrochemically Activated Mononuclear Cu^II^ Complex: A Buffer-assisted, Second-sphere Intermolecular (a) *Versus* A Side-on Intramolecular Pathway (b)

In this context, WO catalysts display a wide variety of ligand architectures. A prominent family of ligands with diverse applications in homogeneous catalysis, including WO, are alkyl-amine macrocycles.^23–25^ These ligands have specific steric and electronic features depending on the cavity size (the number of carbon atoms) or the presence of different substituents (see Scheme 3). For instance, the 14-membered macrocycle with methyl substitution on the amine groups, 14-TMC (14-TMC = 1,4,8,11-tetramethyl-1,4,8,11-tetraazacyclotetradecane), was experimentally^26^ and theoretically^27^ studied toward electrocatalytic WO, yielding an overpotential of 810 mV and a TOF of 30 s^–1^ using Cu­(II) complexes. The effects of ring shrinkage to 12-TMC (12-TMC = 1,4,7,10-tetramethyl-1,4,7,10-tetraazacyclododecane) based Cu­(II) complexes have been further investigated computationally.^22^ In 2025, the 12-TMC catalyst was experimentally synthesized based on a nickel­(II) complex, along with pyridine substitution in one of the tertiary amine groups.^28^ Later on, this pyridine-modified 12-TMC ligand, the Me_3_Pyclen (Me_3_Pyclen = 4,7,10-trimethyl-1,4,7,10-tetraaza-2,6-pyridinophane), was also applied to a copper­(II) complex,^29^ yielding a moderate overpotential (550 mV) and a significant TOF of 66.51 s^–1^ for WO. Although a WO catalytic cycle was proposed with the possibility of single water coordination and noninnocent behavior of the pyridine group based on electrochemical experimental findings, a comprehensive mechanistic study of the electrocatalytic cycle remains pending.

**Scheme 3:**
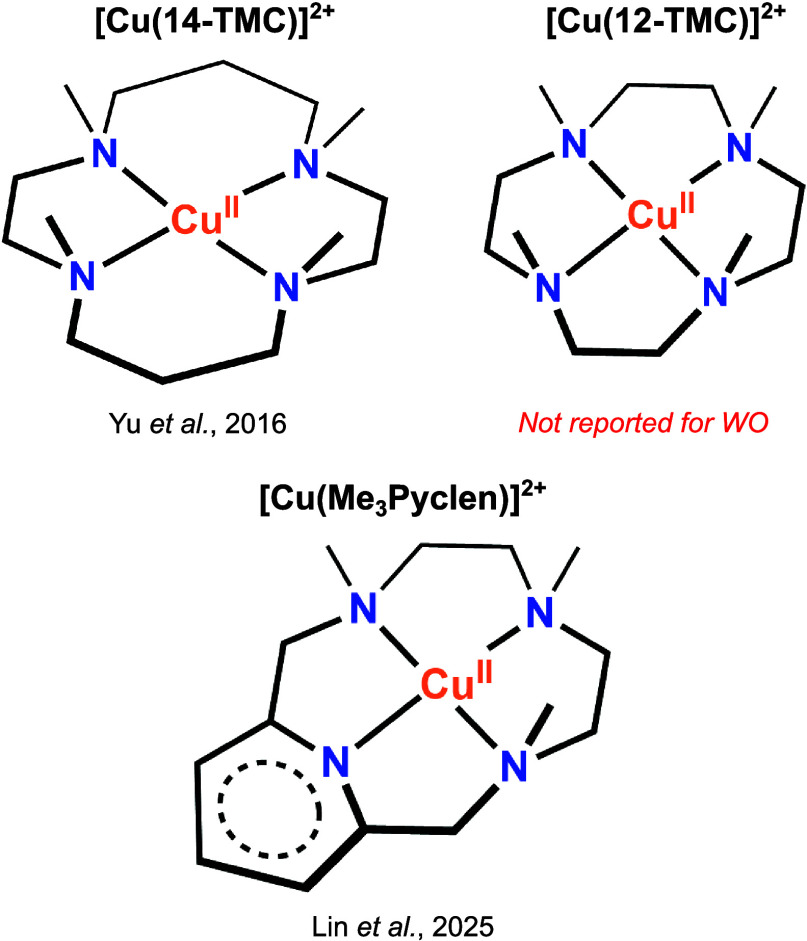
Bidimensional Representations of Different Macrocyclic Copper­(II) Complexes Applied in Electrocatalytic WO

As detailed above, ligand design is a key aspect for improving electrocatalytic WO performance. In this regard, theoretical investigations have been shown to be pivotal for elucidating the electronic and structural factors that govern catalyst activity.^30,31^ For this reason, the present work provides an in-depth computational analysis of the complete catalytic cycle of WO catalyzed by the [Cu­(Me_3_Pyclen)]^2+^ complex using Density Functional Theory (DFT) calculations. Specifically, we explore the impacts of the pyridine-embedded ligand framework based on its electronic aspects, such as backbonding and electron-withdrawing effects, as well as its noninnocent properties. In light of these ligand features and the experimental findings, several Cu-containing reaction intermediates of the elementary steps of the catalytic cycle were investigated along with their energy profiles.^32,33^ By comparing the structural and electronic features of the macrocyclic complexes, it was possible to analyze the kinetics and thermodynamics of the [Cu­(Me_3_Pyclen)] catalysis against previous computational findings of the [Cu­(12-TMC)]^2+^ catalysis, shedding light on the similarities and differences between both electrocatalytic activities. Taken together, the present study aims to provide a comprehensive mechanistic framework to contribute to the design of novel macrocyclic ligands toward more efficient Cu-based WO catalysis.

## Methods

### Electronic Structure Calculations

All stationary points discussed for the WO catalysis were obtained utilizing DFT calculations performed with the Gaussian 09 package.^34^ The optimized structures, along with their harmonic vibrational frequencies, were obtained with the B3LYP functional,^35^ employing the Ahlrichs basis set def2-SVP^36^ for all atoms, except for copper, whose inner-electron relativistic effects were taken into account using the effective-core potential basis set LANL2TZ­(f).^37,38^ The electronic energies were further refined via single-point calculations with the M06L functional^39^ and a larger basis set for the nonmetal atoms (def2-TZVP), while copper was treated using the LANL2TZ­(f) basis set. The saddle points (transition states (TS)) were localized utilizing the Berny algorithm^40^ using the default parameters of Gaussian 09. Occasionally, we performed the quasi-Newton method^41^ for selected TSs. The TS was first supported by the presence of only one imaginary frequency, and further confirmed by intrinsic reaction coordinate (IRC) calculations^42^ for each reaction pathway described herein. In addition, all DFT calculations included Grimme’s empirical dispersion correction D3,^43^ which empirically accounts for energy contributions from van der Waals interactions. The solvation effects from the aqueous bulk environment were simulated using Truhlar’s universal solvation model SMD.^44^ The computational methodology employed herein has been utilized for studying WO electrocatalysis catalyzed by similar macrocyclic copper­(II) complexes,^27,45^ providing good correspondence with experimental data and supporting our methodological choice. In order to further validate the DFT level described above for [Cu­(Me_3_Pyclen)]^2+^ catalysis, a short benchmarking study was also carried out with other functionals, likewise providing good agreement with experimental data (see details in the Supporting Information, SI, Table S1).

For electronic distribution and charge transfer elucidation, spin density isosurfaces and its atomic values were obtained through the Multiwfn software,^46,47^ along with Hirshfeld charges.^48^ Giving the propensity for the occurrence of minimum energy crossing points (MECP) between potential energy surfaces (PES) of different spin multiplicities in 3*d* metal complexes,^49^ we employed the Harvey algorithm for MECP search as implemented in the EasyMECP code.^50^ Broken-symmetry calculations for open-shell singlet species were performed in the Orca 6.0 package^51^ with the same DFT level of theory, in order to simulate a spin inversion on a localized center of the molecular system. In this case, following the Heisenberg–Dirac-Van Vleck formalism (eq 2), which captures spin correlations in multielectron systems through an effective Hamiltonian, the widely used Yamaguchi method (eq 3)^52,53^ was applied to derive the exchange coupling constant *J*_*AB*_ between two molecular sites, A and B, by relating the energy difference between the high-spin and broken-symmetry states to the effective spin–spin coupling

2 $${Ĥ}_{\mathrm{HDVV}}=-2J_{\mathrm{AB}}S_{A}\cdot S_{B}$$

3 $$J_{\mathrm{AB}}=-\frac{E_{\mathrm{HS}}-E_{\mathrm{BS}}}{\langle S^{2}\rangle_{\mathrm{HS}}-\langle S^{2}\rangle_{\mathrm{BS}}}$$

in which *E*_HS_ and *E*_BS_ are the electronic energy of the high-spin and broken-symmetry systems, respectively, and the calculated expected values for spin angular momentum squared operator in high-spin and broken-symmetry systems are respectively ⟨*S* ^2^⟩_HS_ and ⟨*S* ^2^⟩_BS_. In summary, positive values for *J*_*AB*_ indicate ferromagnetic coupling, whereas negative values indicate antiferromagnetic coupling.

### Thermodynamics

The Gibbs free energy values of all stationary points at 298.15 K in standard solution *G*_*i*_^°^ were derived from the eq 4, for which the Phyton code Goodvibes^54^ was used to compute quasi-harmonic corrections to the vibrational entropy with a frequency cutoff value of 100 cm^–1^, based on Grimme’s model^55^

$$G_{i}^{{}^{\circ}}\left(\mathrm{aq}\right)=E_{i}+\mathrm{qh}‐G_{i}^{\mathrm{corr}}+\mathrm{RT}\;\ln\left(\frac{\mathrm{RTc}}{p}\right)$$ 4

wherein *E*_*i*_ is the electronic energy; *qh*-*G*_*i*_^*corr*^ is the quasi-harmonic thermal correction to *G* at 298.15 K and 1 atm (both from calculations with implicit solvation); and the last term is the concentration correction from *p* = 1 atm to *c* = 1 M, whose value is approximately 1.89 kcal/mol. Specifically for the water, a concentration of 55.56 M was taken, resulting in a correction of 4.27 kcal/mol.

Regarding outer-sphere electron transfer events, the energy barriers of these processes were calculated by the Marcus Theory formalism,^56–58^ as depicted in the Section S2 of Supporting Information. For electrochemical processes, the standard redox potentials *E*_O|R_^°^ (vs SHE) were calculated from

5 $$E_{O|R}^{{}^{\circ}}=\frac{G_{O}^{{}^{\circ}}\left(\mathrm{aq}\right)+n_{H^{+}}G_{H^{+}}^{{}^{\circ}}\left(\mathrm{aq}\right)-G_{R}^{{}^{\circ}}\left(\mathrm{aq}\right)}{n_{e}F}-E_{\mathrm{SHE}}^{{}^{\circ}}$$

for which the Gibbs free energy of the proton in aqueous solution *G*_H^+^_^°^(aq) was considered as – 270.3 kcal/mol,^59^ the absolute value for the standard hydrogen electrode *E*_SHE_^°^ was taken as 4.281 V^60^ both thermodynamic values obtained from experimental data. The Faraday constant *F* was given by 23.08 kcal/mol. Also, the Gibbs free energies *G*_O_^°^(aq) and *G*_R_^°^(aq) correspond to the oxidized and reduced species in aqueous solution, respectively. In proton-coupled electron transfers, the number of protons *n*_H^+^_ involved is different from zero; hence there’s a pH dependency on the redox potential. In this sense, the Nernst equation was applied in order to mimic the experimental conditions (pH = 7)

6 $$E_{O|R}=E_{O|R}^{{}^{\circ}}-\frac{n_{H^{+}}}{n_{e}}\frac{\mathrm{RT}}{F}\ln\left(10\right)\mathrm{pH}$$

In such processes, it is also interesting to estimate the p*K*_a_ relative to deprotonation steps by the following expression

7 $${pK}_{a}=\frac{G_{A^{-}}^{{}^{\circ}}\left(\mathrm{aq}\right)+n_{H^{+}}G_{H^{+}}^{{}^{\circ}}\left(\mathrm{aq}\right)-G_{\mathrm{AH}}^{{}^{\circ}}\left(\mathrm{aq}\right)}{\mathrm{RT}\;\ln\left(10\right)}$$

where *G*_AH_^°^(aq) and *G*_A^–^_^°^(aq) correspond to the standard Gibbs free energies of the solvated protonated and deprotonated species, respectively.

## Results and Discussion

### Electrochemical Generation of Active Species

Our first attempts regard the importance of establishing the most stable conformer of the complex [Cu­(Me_3_Pyclen)]^2+^ in aqueous solution,^61^ in order to find a representative catalytic structure. To this end, a short analysis of three possible structures was carried out (see Supporting Information, Figure S2a), arising from torsions in two nonrigid C–C bonds of the macrocycle. Afterward, we explored the electrochemical activation of the catalyst by scrutinizing the water-coordinated complex ^2^ **1**, for which the quartet multiplicity lies energetically prohibitive (see Supporting Information, Figure S2b). Following a PCET computed to be 1.35 V from the catalytic precursor (as shown in Figure 1), the triplet species ^3^ **2** is formed smoothly, with the singlet analogue lying 1.02 kcal/mol higher in Gibbs free energy. It is worth mentioning that we have explored several isomeric forms of all structures depicted in Figure 1 in order to establish the most stable one (see Supporting Information, Figure S2). In addition, all spin density values obtained with M06L-D3/Def2-TZVP level of theory are detailed in Supporting Information, Table S1. The spin density analysis shows that the electron loss is associated with the Cu–L moiety (L = Me_3_Pyclen), with a slight contribution from the oxygen. The subsequent electrochemical activation steps indicated by cyclic voltammetry (CV) correspond to a SET process with a redox potential of 1.39 V and a PCET with a redox potential of 1.63 V, even though it is not possible to determine their order of occurrence by the calculations. In this sense, we decided to state both scenarios as equally plausible in the catalytic cycle.

**Figure 1:**
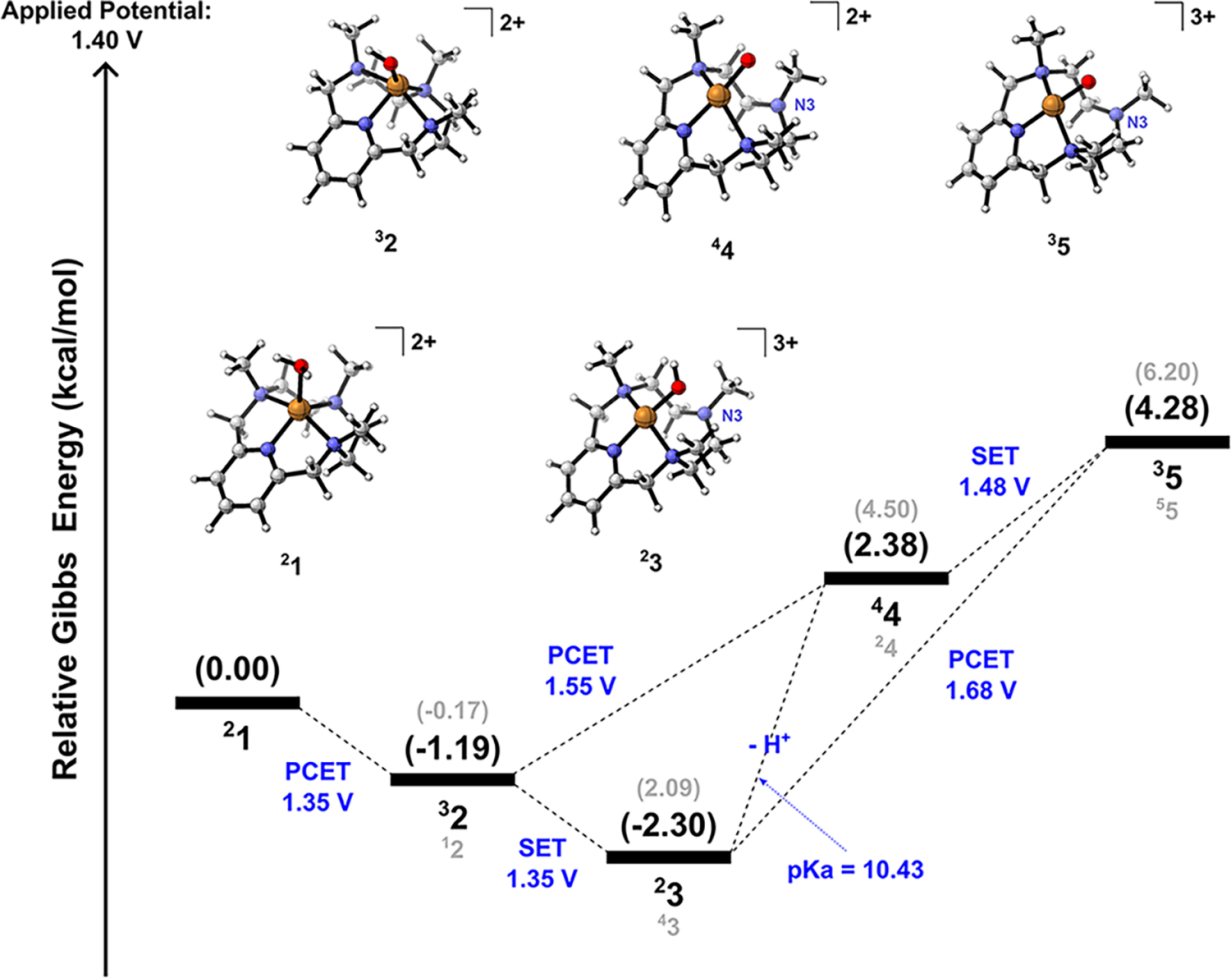
Gibbs free energy profile of electrochemical activation of the catalyst [Cu­(Me_3_Pyclen)­H_2_O]^2+^ (^2^ **1**) considering a single-coordinating water pathway.

Regarding the SET (1.35 V), the doublet species ^2^ **3** is also formed smoothly, with its corresponding quartet species computed to be 4.39 kcal/mol higher in energy. The structure of ^2^ **3** shows an uncoordinated nitrogen holding an unpaired electron, as further demonstrated by the spin densities. Furthermore, the product of this SET is a Cu^III^ complex (*d* ^8^ configuration). In sequence, the calculations show that an exergonic PCET of 1.68 V leads to ^3^ **5**, for which the quintet analogue is less stable by 2.92 kcal/mol, while maintaining a similar coordination sphere environment. Likewise, according to the spin densities of ^3^ **5**, this oxidation process from PCET does not involve the copper ion. Instead, the oxidation occurs predominantly on the oxygen. In the opposite trend, when the oxidation starts from a PCET (1.55 V), (^3^ **2** → ^4^ **4**), the tendency of N-uncoordination is revealed by the calculations, with the high-spin configuration being more stable by 2.22 kcal/mol. In contrast with the SET scenario, spin density analysis shows that the metal center remains as Cu^II^ (*d* ^9^ configuration). In this sense, the PCET transformations involve predominantly the oxygen rather than the Cu center. Afterward, a SET (1.48 V) takes place, leading to ^3^ **5**, thereby oxidizing the metal center from Cu^II^ to Cu^III^. Interestingly, broken-symmetry calculations show no antiferromagnetic coupling (see Supporting Information, Table S3), such that the open-shell singlet species ^1^ **5** is less stable than its triplet analogue ^3^ **5**. Importantly, all redox potential values reported in the experimental work are in very good agreement with our computations,^29^ thus supporting that these mechanistic scenarios for the electroactivation of the catalyst are reasonable.

In order to expand the mechanistic possibilities, we also explored another reaction route arising from the possibility of double water coordination (i.e., two water molecules acting as ligands in species ^2^ **1**). Unfortunately, this was not successful, since the optimization process tended to dissociate one water molecule from the complex, thus maintaining its pentacoordinate environment. On the other hand, considering the second water binding from ^3^ **2**, this coordination leads to the hexacoordinated complex ^3^ **2**′ with a distorted octahedral geometry; the process is slightly energetically favorable by 1.31 kcal/mol (Figure 2). In this direction, we also decided to test various isomers for these species with double water coordination (see Supporting Information, Figure S3), as well as possible ligand configurations related to the two water ligands, e.g., an inversion between the relative positions of the two oxygen atoms in the complex (see Supporting Information, Figure S4). As a result, the sequence of electrochemical events may involve proton release from either oxygen atoms, i.e., from O1 or O2 of the water ligands. For instance, regarding the O1-centered sequence of intermediates, we used a prime (′) superscript to identify the complexes formed from O1, while those formed from the O2-centered sequence are labeled with double prime superscripts (″). It is possible to observe from the calculations that the intermediates ^4^ **4**′ and ^4^ **4**″ preserve the octahedral geometry. On the other hand, ^2^ **3**′ and ^3^ **5**′ complexes adopt a square pyramidal geometry with one uncoordinated nitrogen. The last species ^3^ **5**′ is more stable than the broken-symmetry singlet analogue, while ^3^ **5**″ appears to be almost isoenergetic (see Supporting Information, Table S3). We point out that these alternative mechanisms for SETs and PCETs also reproduce the experimental redox potential values, in such a way that three pathways may arise for the O–O bond formation, showing how intricate the reaction mechanism of the electrocatalytic cycle can be in this first sequence. For this reason, the calculations indicate the species ^3^ **5**, ^3^ **5**′, and ^3^ **5**″ as reliable precursor complexes for the O–O bond formation, as will be discussed in the following section.

**Figure 2:**
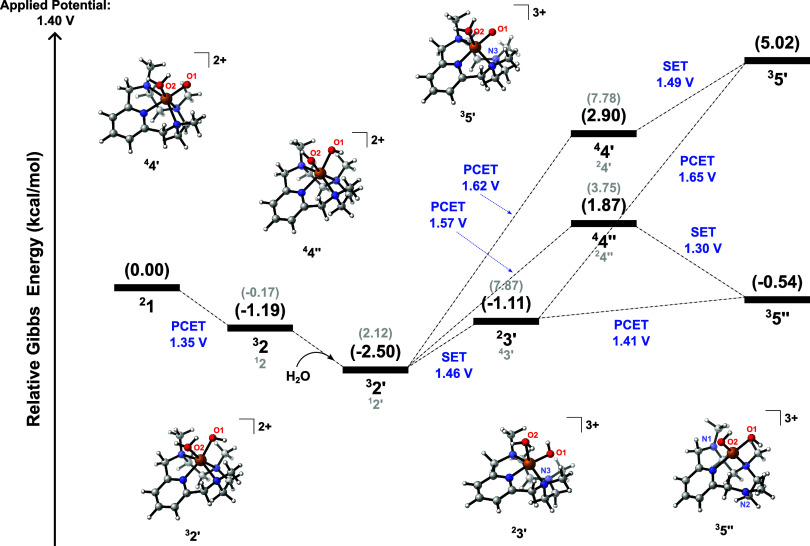
Gibbs free energy profile of electrochemical activation of the catalyst [Cu­(Me_3_Pyclen)­H_2_O]^2+^ (^2^ **1**) considering a double-coordinating water route.

### The O–O Coupling Mechanisms

Considering first the O–O coupling pathway from ^3^ **5**, in which the electrochemical activation involves only one water molecule, the addition of the buffer species HPO_4_^2–^ along with another water molecule leads to the reactive complex ^3^ **RC**_5_ (Figure 3a). It can be noted from its spin density isosurface (Figure 3b) that, when the phosphate anion approaches the vicinity of the complex, an electron is donated via an outer-sphere electron transfer with an energy barrier computed to be 11.68 kcal/mol (**OS-SET**_5_). As a result, the copper center shifts its oxidation state from Cu^III^ (in species ^3^ **5**) to Cu^II^ (see Supporting Information, Table S2). Specifically, in this outer-sphere electron transfer mechanism, the ion HPO_4_^2–^ holds an unpaired electron delocalized in a π orbital. It is worth mentioning that Figure S6 displays additional spin density isosurfaces at the B3LYP-D3/Def2-SVP level of theory regarding key species involved in O–O bond formation; nevertheless, M06L-D3/Def2-TZVP still correctly describes the following mechanisms. Additionally, we examined the feasibility of a broken-symmetry singlet from ^3^ **RC**_5_, in which ferromagnetic coupling was evidenced (see Supporting Information, Table S3). Moreover, considering that ^3^ **RC**_5_ displays a total of four unpaired electrons, we investigated an analogous quintet state that potentially may be accessed via a MECP (see Supporting Information, Figure S5a), perhaps due to spin inversion of a β electron. However, after several attempts, no TS was found related to the O–O coupling at quintet multiplicity. Considering both scenarios, we opted to focus on the triplet PES throughout the discussion of the O–O coupling step of the catalytic cycle.

**Figure 3:**
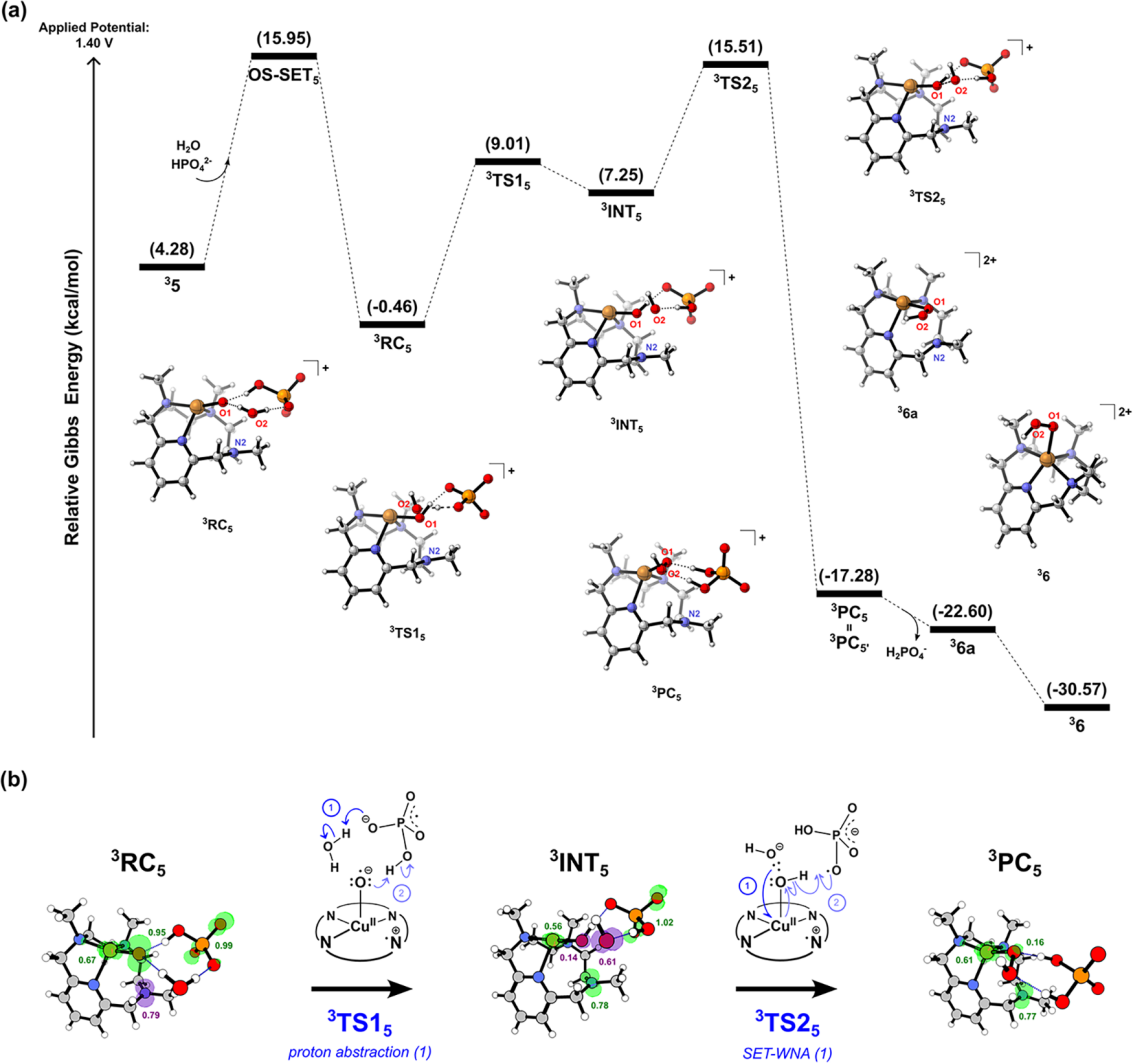
(a) Gibbs free energy profile for the O–O bond formation via Pathway ^3^ **5** and (b) Spin densities isosurfaces (obtained at the M06L-D3/Def2-TZVP level of theory) and bidimensional structures of key intermediates for rationalizing the mechanism.

In sequence, ^3^ **RC**_5_ undergoes proton abstraction from water by the phosphate anion, with a TS identified (^3^ **TS1**_5_). Interestingly, the IRC calculation indicated that, prior to this process, another concerted proton transfer occurs, this time from the buffer species to the oxygen coordinated to the complex (Figure 3b). We therefore presume that a double proton exchange by the phosphate anion takes place, maintaining its charge state of −1. As a result, the intermediate ^3^ **INT**_5_ is formed, wherein both oxygen atoms interact frontally, and it is observed that the unpaired electron is more localized on the O2 atom. ^3^ **INT**_5_ is, thereby, the precomplex from which the O–O coupling proceeds. The calculations indicate a SET-WNA mechanism (^3^ **TS2**_5_) leading to ^3^ **PC**_5_ as the product. In this SET process, an electron is transferred from the three-electron interaction between both oxygen atoms,^62–64^ resulting in a shift from Cu^II^ to Cu^I^ (see Supporting Information, Figure S7, in which this process is supported by the spin density isosurfaces of the TSs). Indeed, the IRC confirms that, after O–O bond formation, a hydrogen atom is concertedly transferred from oxygen O1 to the radical anion HPO_4_^•–^, thereby restoring, at the end of the O–O coupling, the Cu^II^ complex and the closed-shell species H_2_PO_4_^–^ in ^3^ **PC**_5_. This hydrogen atom transfer (HAT) mechanism via ^3^ **TS2**_5_ → ^3^ **PC**_5_ is consistent with Hirshfeld charge analysis (see Supporting Information, Table S4), since there is no significant variation in the charge of oxygen O1. Additionally, the charge fluctuation at the metal center agrees with the electron transfer to Cu^II^ under discussion (see details in Figure 3b).

Overall, considering the O–O bond coupling and the outer-sphere SET reaction steps computed for the catalytic cycle, we find that the energy barriers associated with both pathways are in good agreement with the experimentally measured TOF (which implies a Δ*G* ^‡^ of approximately 15.02 kcal/mol at 298.15 K). Based on the calculations, it is not possible to single out which step is the TOF-determining step. For instance, the O–O bond coupling was estimated to be 15.97 kcal/mol by the energy span model,^65,66^ considering ^3^ **RC**_5_ as the TOF-determining intermediate (TDI). Furthermore, we explored an alternative pathway from ^3^ **5** in which the complex structure displays N3 as the uncoordinated nitrogen, instead of N2 (see Supporting Information, Figure S7). Unfortunately, the reaction path for this O–O coupling TS was not conclusive after IRC calculation, i.e., it was not possible to establish chemical connectivity between the intermediates and the TS related to the mechanism presented herein, even though the energy barrier was computed to be quite similar.

Regarding the pathway ^3^ **5**′, the calculations have shown the same tendency for outer-sphere electron transfer, in which it is possible to observe that, when the HPO_4_^2–^ anion is added to the system, the reactive complex ^3^ **RC**_5′_ is formed (Figure 4a), resulting once again in the shift of the copper oxidation state from Cu^III^ to Cu^II^ and leading to the radical phosphate anion HPO_4_^•–^ (see details in Figure 4b). In this case, the calculated Marcus theory barrier for this outer-sphere electron transfer is slightly higher (13.39 kcal/mol). Similarly, the broken-symmetry counterpart was investigated, resulting in ferromagnetic coupling (see Supporting Information, Table S3). Furthermore, MECPs connecting the surfaces were revealed to access the quintet PES (see Supporting Information, Figure S5b). Once again, for the current reaction route involving O–O coupling, the TS on the quintet state was not found, in spite of several attempts.

**Figure 4:**
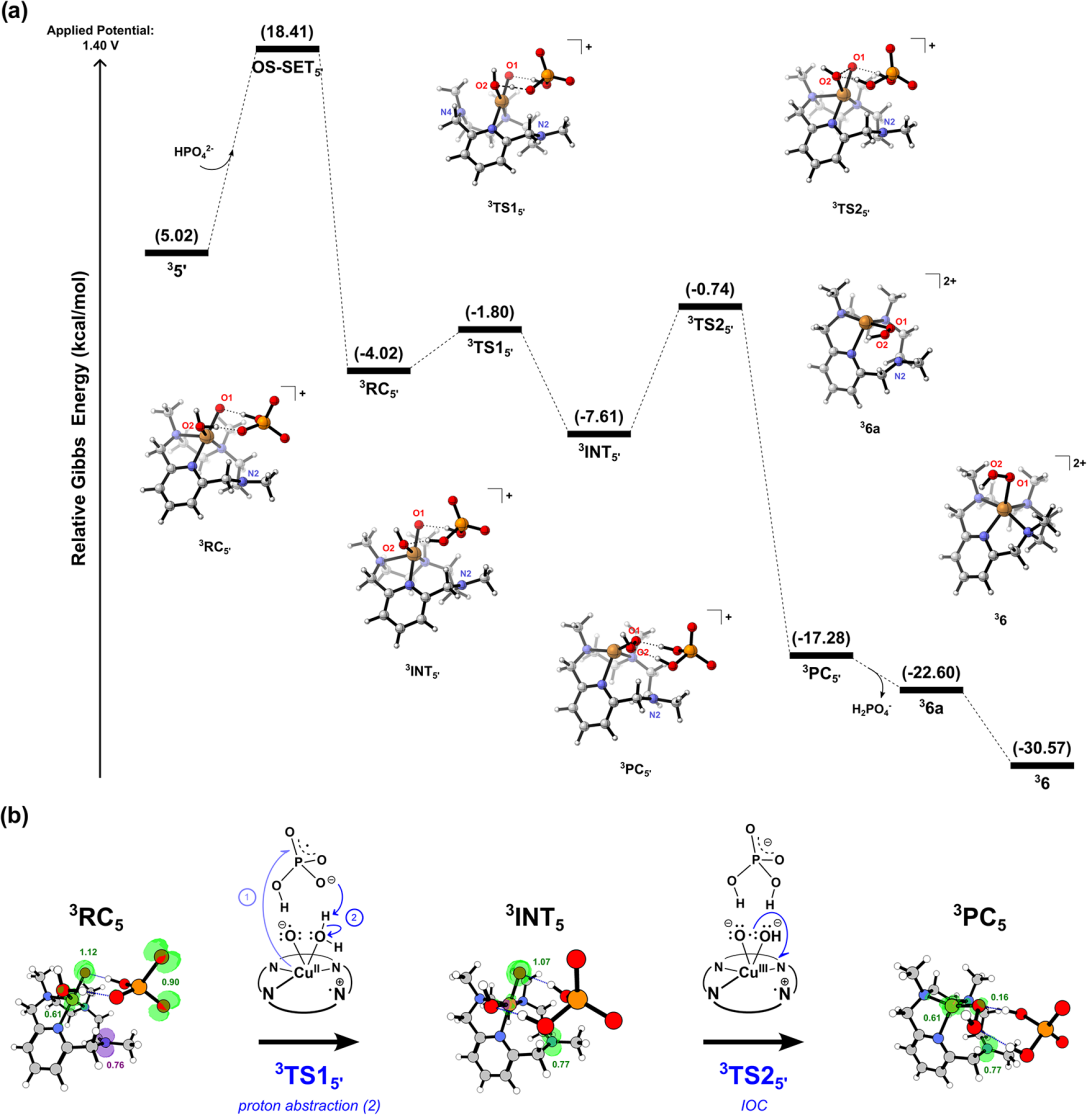
(a) Gibbs free energy profile for the O–O bond formation via Pathway ^3^ **5**′ and (b) Spin densities isosurfaces (obtained at the M06L-D3/Def2-TZVP level of theory) and bidimensional structures of key intermediates for rationalizing the mechanism.

On the other hand, on the triplet PES, an energetically feasible proton transfer process was revealed by the calculations from oxygen O2 (i.e., from the coordinated water) to the HPO_4_^•–^ species, as indicated by the imaginary frequency of ^3^ **TS1**_5′_, leading to the intermediate ^3^ **INT**_5′_ as the product. Interestingly, the IRC calculations show that, before the preceding migration, an electron is donated from the complex to the phosphate moiety, which thus shifts from HPO_4_^•–^ to HPO_4_^2–^. As a result, the coordination mode of the complex changes, promoting a transient N4 dissociation from the metal center (see details from the spin density isosurfaces in Figures 4b and S8). In summary, the chemical transformation regarding ^3^ **RC**_5′_ → ^3^ **INT**_5′_ involves an oxidation state shift from Cu^II^ to Cu^III^ at the metal center. In addition, during the proton migration, the HPO_4_^2–^ anion becomes H_2_PO_4_^–^.

Afterward, the O–O coupling occurs via an IOC mechanism. Indeed, the imaginary frequency of ^3^ **TS2**_5′_ has been associated with the ∠O1–Cu–O2 deformation (bending) that leads to species ^3^ **PC**_5′_, which is the same stationary point as ^3^ **PC**_5_, as confirmed by IRC calculations. In this case, in order to form the O–O bond, the calculations reveal that an electron is transferred from the repulsive three-electron O–O interaction to the metal center. Therefore, a transition from Cu^III^ to Cu^II^ is observed (see details in Figure 4b). As expected, the computed energy barrier for this IOC mechanism is 6.87 kcal/mol, which is considerably lower than the reaction barrier computed for the SET-WNA pathway. However, the most significant energetic hurdle for the overall pathway from ^3^ **5**′ consists of the outer-sphere electron transfer reaction barrier estimated by Marcus Theory.

Finally, the HPO_4_^2–^ anion departs from the system, thus forming intermediate ^3^ **6a**, in which nitrogen N2 remains uncoordinated. However, a restoration of the pentacoordinate geometry is observed for species ^3^ **6** (i.e., with N2 coordination), producing significant stabilization (this occurs in a similar fashion for the pathway from ^3^ **5**). It is worth mentioning that we have also examined another possible reaction pathway involving the prereactive complex ^3^ **5**″ (see Supporting Information, Figure S9). Although in this case the TS for the O–O coupling could not be located, it is possible that, after the formation of a reactive complex upon inclusion of HPO_4_^2–^ in the system (^3^ **RC**_5″_), hydroxide dissociation from the complex may lead to ^3^ **INT**_5_, thus producing a crossing of pathways (i.e., the pathway from ^3^ **5**″ connects with the pathway from ^3^ **5**).

Once the O–O bond has been formed, the intermediate ^3^ **6** may undergo another PCET in order to generate molecular oxygen O_2_. In this case, we investigate two possibilities: (1) a deprotonation followed by a SET, or (2) an inverse sequence: a SET followed by a deprotonation. Both mechanistic scenarios are illustrated in Figure 5. As discussed, the PCET may occur through a proton loss (^3^ **7**) and a SET leading to ^4^ **9**, since both steps are considerably exergonic. As expected, the electron loss is oxygen-centered, instead of involving the metal center, which remains at the oxidation state Cu^II^ (see details in the Support Information, Table S2). Finally, a substitution of coordinated O_2_ (at triplet state) and a water molecule restores the original doublet catalyst ^2^ **1**. At this point, the catalytic cycle restarts.

**Figure 5:**
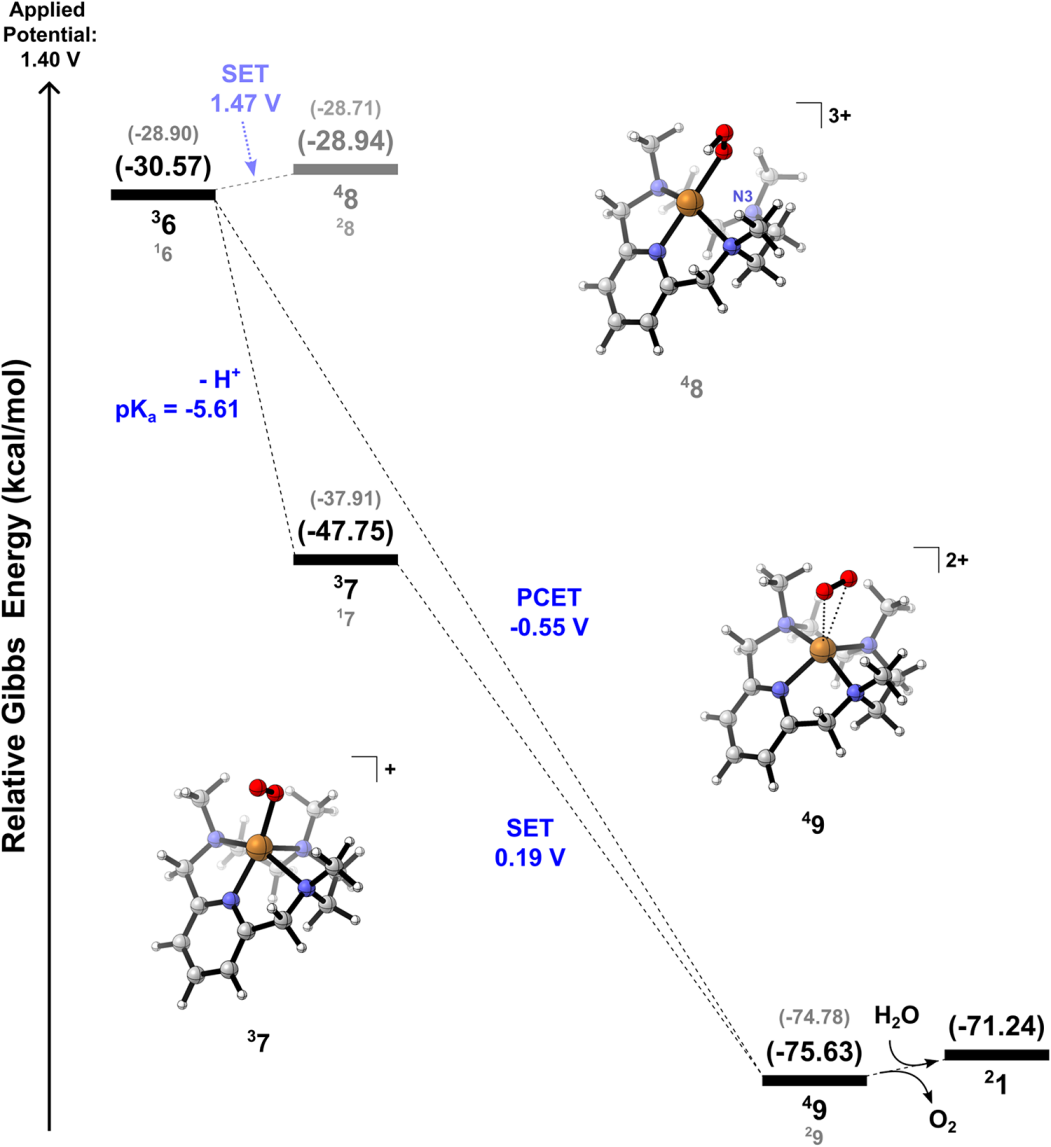
Gibbs free energy profile of the O_2_ formation and its departure from the system, which thus restores the original catalyst.

### The Pyridine Effect: [Cu­(Me_3_Pyclen)] vs [Cu­(12-TMC)]

In this section, we decided to compare two distinct macrocycles (Scheme 3), both being candidate catalysts: [Cu­(12-TMC)]^2+^ and [Cu­(Me_3_Pyclen)]^2+^, which differ in their macrocyclic architectures, i.e., due to the modification of a tertiary amine into a pyridine. This modulation leads to key structural and electronic changes that are reflected in their respective catalytic energy profiles and, by extension, in their kinetic and thermodynamic parameters. Starting from the electrochemical activations with a single coordinated water molecule (see Supporting Information, Figure S10), it is possible to observe that, for the Me_3_Pyclen ligand, the first two electron losses are thermodynamically more feasible, while the third electron loss is more energetically demanding. This tendency can be rationalized through key molecular orbitals (MOs) of species ^2^ **3** (Figure 6). It has been noted that the Me_3_Pyclen ligand is strongly influenced by π donation from the pyridine moiety when compared with its 12-TMC analogue ^4^ **C**, to the point of shifting from high-spin to low-spin multiplicity.

**Figure 6:**
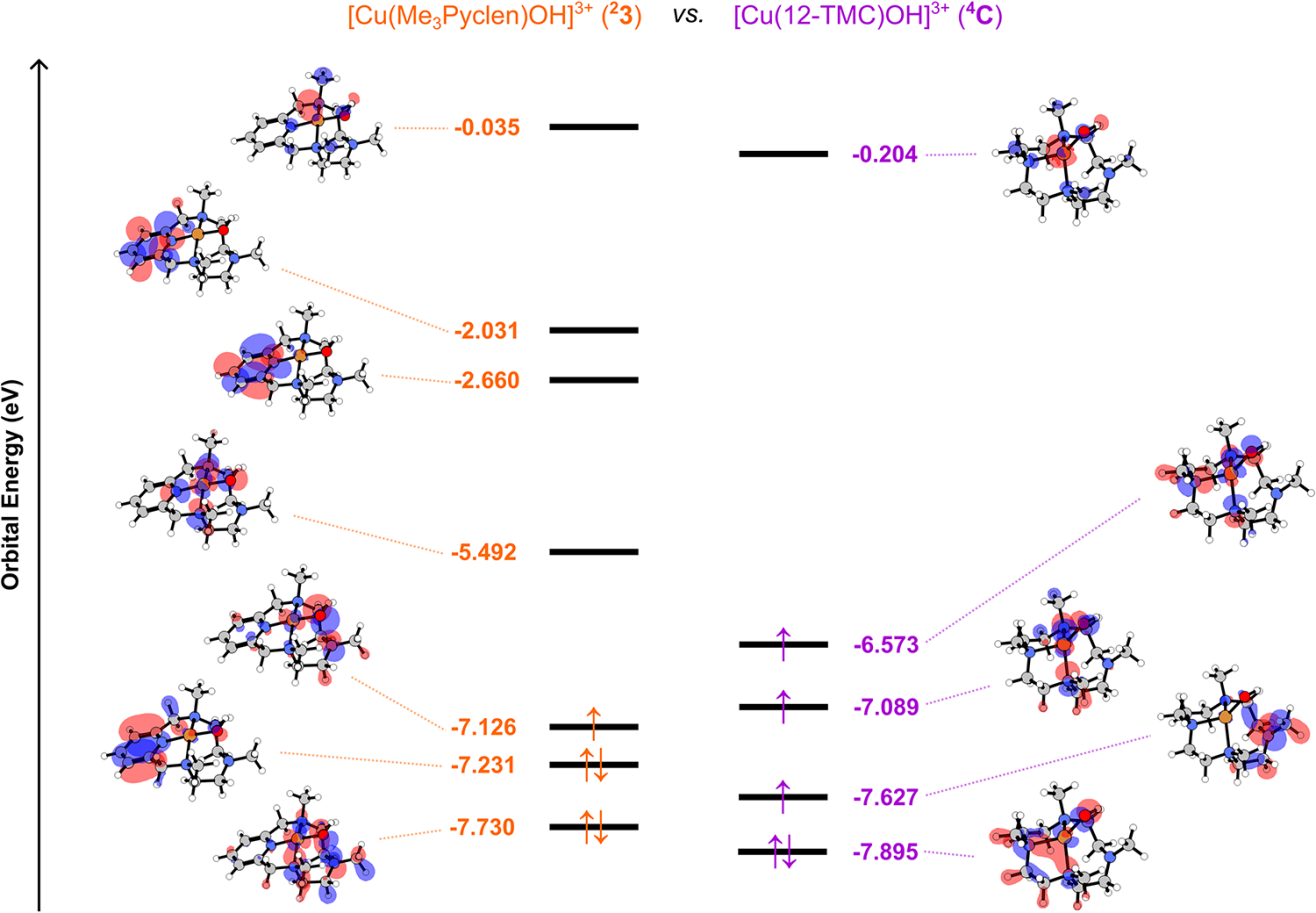
Molecular orbital diagrams of the analogue species ^2^ **3** (with Me_3_Pyclen ligand) vs ^4^ **C** (with 12-TMC ligand), both obtained at the M06L-D3/Def2-TZVP level of theory.

In this case, it is also noted that such a combination between conjugated orbitals of the aromatic ring and specific *d* orbitals of the metal produces lower orbital energies compared with those from 12-TMC. In addition, the MOs of Me_3_Pyclen are close in energy. We believe that this MO picture induces electron pairing (doublet configuration) for Me_3_Pyclen. Accordingly, it is expected that more energy is required to remove a single electron from ^2^ **3** when compared to its 12-TMC analogue. However, it is important to point out that this π backbonding effect should not be confused with ligand noninnocence,^67^ as previously proposed by the experimental group.^29^ The lack of spin density (unpaired electrons) associated with the pyridine moiety is a strong indication that Me_3_Pyclen is not oxidized or reduced during catalyst activation. Instead, it is the effects of charge transfer via orbital combination that govern the differences between both electronic structures and, therefore, the thermodynamic parameters involved in each catalytic system.

With respect to the structures of the key complexes, as shown in the Supporting Information (Figure S11), subtle geometric discrepancies have been observed that arise from the pyridine substitution. Despite the general similarities in the coordination environments of each analogue intermediate, it is possible to notice that complexes based on the Me_3_Pyclen ligand show a tendency toward planarity among the coordinated atoms, produced by the delocalization of π-like MO combinations from the pyridine group. For instance, using the structures from Figure 6, it becomes evident that the tetracoordination geometry of ^2^ **3** is almost square planar, while for ^4^ **C**, it resembles a distorted tetrahedron. The same tendency is repeated in all tetracoordinated species (see these structures in Supporting Information, Figure S11, and the specific angles reported in Table S5). This structural deformation is one of the factors that helps explain the feasibility of a second water coordination to the complex with the Me_3_Pyclen ligand in the axial position, resulting in a square pyramidal or distorted octahedral geometry, depending on the coordination number of the complex. Another important aspect observed from the calculations of the pyridine-embedded macrocycle is the reduced steric hindrance compared to the tertiary amine bearing a methyl group, thus providing a unique steric environment and, consequently, influencing the coordination mode of the complex (i.e., the viability of double water coordination). For this reason, the mechanism of O–O bond formation with the Me_3_Pyclen ligand proceeds more easily. For example, the energy barrier for the SET-WNA pathway with the 12-TMC ligand is 19.26 kcal/mol (vs 15.97 kcal/mol), whereas for the IOC pathway, the barrier is 14.13 kcal/mol (vs 6.87 kcal/mol).^22^

## Conclusions

In summary, we performed DFT calculations to explore the electrocatalytic cycle involving the pyridine-modified 12-TMC ligand, Me_3_Pyclen (Me_3_Pyclen = 4,7,10-trimethyl-1,4,7,10-tetraaza-2,6-pyridinophane), based on Cu­(II) complexes. It was possible to identify two possible mechanisms for the O–O bond formation: a single-coordination SET-WNA and a double-coordination IOC; both pathways are computed to be viable at room temperature and reproduce well the experimental TOF. Furthermore, consistent with previous experimental and theoretical studies, our calculations support the IOC as a lower-barrier process, although the preceding outer-sphere electron transfer might also play an important role in the catalytic efficiency, as well as in generating reactive complexes that promote the buffer-mediated O–O coupling. Additionally, it is also important to emphasize the electronic and steric features revealed by the calculations on the pyridine-embedded ligand, which might be relevant for the design of new macrocyclic ligands for WO. In conclusion, our energetic, structural and electronic findings from theoretical investigations of electrocatalytic WO with the complex [Cu­(Me_3_Pyclen)]^2+^ as electrocatalyst provide a comprehensive picture of its mechanistic features, highlighting how ligand modulation governs key molecular properties and impacts catalytic performance. Furthermore, this work also paves the way for the rational functionalization of pyridine via computational tools toward enhanced WO catalysis promoted by Cu complexes.

## Supplementary Material





## Notes

Cartesian coordinates of all stationary points of the main text, along with their respective SCF energies (XYZ)
Computational data (PDF)
