# Supplementary material for: Macrocyclic Copper(II) Electrocatalysts for Water Oxidation: Catalytic Mechanism and Activity of Pyridine-Embedded Complexes
Source: J Phys Chem A. 2026 Jun 23;130(26):4930–41. doi: 10.1021/acs.jpca.6c01912 (PMC13339654; doi:10.1021/acs.jpca.6c01912)
Supplement: Supplementary file 2 [file jp6c01912_si_002.pdf]

Supporting Information

Macrocyclic Copper(II) Electrocatalysts for Water Oxidation: Catalytic Mechanism and Activity of Pyridine-Embedded Complexes

João Pedro C. S. Neves†, Roberto Rivelino‡, Tiago Vinicius Alves*,¶ and Vitor H. Menezes da Silva*,¶

†*Departamento de Físico-Química, Instituto de Química, Universidade Federal da Bahia, Rua Barão de Jeremoabo, 147, Salvador, Bahia, 40170-115, Brazil*

‡*Instituto de Física, Universidade Federal da Bahia, Salvador, Bahia 40210-340, Brazil*

¶*Departamento de Química, Centro de Ciências Exatas, Universidade Estadual de Londrina, Rodovia Celso Garcia Cid, PR 445 Km 380, Londrina, 86050-482, Paraná, Brasil*

*E-mail address: tiago.alves@uel.br, vmenezesdasilva@uel.br

Sections

S1. Benchmarking S3
S2. Marcus Theory Calculations S4
S3. Alternative Structures S6
S4. Spin Densities S9
S5. Broken-Symmetry Calculations S11
S6. Additional Information on O−O Bond Formation S12
S7. Additional Information on 12-TMC Comparison S16

Tables

S1. Benchmarking of the first PCET redox potentials comparing DFT values with respect to the experimental reference of 1.29 V, with their respective relative errors (RE). S3
S2. Calculated spin densities (M06L-D3/Def2-TZVP) of the investigated species from main text. S9
S3. Broken-symmetry data for selected triplet species S11
S4. Charge transfer analysis of selected intermediates and TSs employing Hirshfeld charge (q_H) calculations. S15
S5. Geometric comparison between selected η^3 intermediates of 12-TMC and Me_3Pyclen catalysis, based on Figure S10. . . . . S17

Figures

S1. Marcus Theory energy diagram for outer-sphere electron transfer processes. S4
S2. Alternative structures for the stationary points showed in the Figure 1. . . S6
S3. Alternative structures for the stationary points showed in the Figure 2. . . S7
S4. Alternative structures for the stationary points showed in the Figure 2, switching relative positions of the coordinated oxygens. S8

S5. Gibbs free energy profiles of the Pathways ^{3}**5** (a) and ^{3}**5′** (b) – in grey – considering minimum energy crossing points with the quintet multiplicity surface – in black. S12

S6. Spin density isosurfaces of key species of Figures 3 and 4 of the main text, obtained with B3LYP-D3/Def2-SVP level of theory. S13

S7. Spin density isosurfaces of transition states indicated in Figures 3 and 4 of the main text (obtained with M06L-D3/Def2-TZVP level of theory). S14

S8. Gibbs free energy profiles of the alternative Pathway ^{3}**5**, in which the uncoordinated nitrogen is N3 instead of N2; the IRC of ^{3}**TSb**$_5$ was not conclusive, although the energy barrier is consistent with experimentally measured TOF. S14

S9. Gibbs free energy profile of the Pathway ^{3}**5″**. Regarding O–O bond formation, there was no new TS found; however, upon O2 dissociation from the complex (whose TS also wasn't found), the catalysis may proceed through Pathway ^{3}**5** – in grey color. S15

S10. Gibbs free energy profiles of electrochemical activation for WO with different catalysts: 12-TMC (in red) *vs.* Me_3Pyclen (in black). Both were calculated at the same DFT level of theory. S16

S11. Optimized structures of selected η^3 intermediates for 12-TMC *vs.* Me_3Pyclen comparison. S17

S1. Benchmarking

Table S1.: Benchmarking of the first PCET redox potentials comparing DFT values with respect to the experimental reference of 1.29 V, with their respective relative errors (RE).

**Methodology**	**PCET (V)**	**RE (%)**
B3LYP-D3/Def2-TZVP	1.81	40.31
ωB97X-D/Def2-TZVP	2.17	68.22
M06-D3/Def2-TZVP	2.05	58.91
M06L-D3/Def2-TZVP	1.35	4.65
PBE-D3/Def2-TZVP	1.20	6.98
TPSS-D3/Def2-TZVP	1.24	3.88

Obs.: It is worth mentioning that a better description of this system is obtained using non-hybrid functionals (namely M06L, PBE, and TPSS), whereas the tested hybrid functionals did not properly describe the redox process under consideration. Although the TPSS functional yields the lowest relative error, the M06L results were considered sufficiently accurate, showing only a small deviation relative to TPSS. Moreover, the use of M06L enables a more direct comparison with the catalysis energy profile of $[Cu(12\text{-}TMC)]^{2+}$, which was computed at this level of theory. Therefore, the M06L functional was retained for the $[Cu(Me_3Pyclen)]^{2+}$ catalysis calculations.

S2. Marcus Theory Calculations

The calculations for outer-sphere electron transfer energy barriers were grounded by the formalism described by Rudolph Marcus (references 56-58 of the main text), which has been further refined by several computational methodologies such as, for example, the procedures reported by Batista *et al.* [1] and Maseras [2]. The approach used in this work was recently demonstrated to be appropriate for the context of electrocatalytic water oxidation with copper complexes [3].

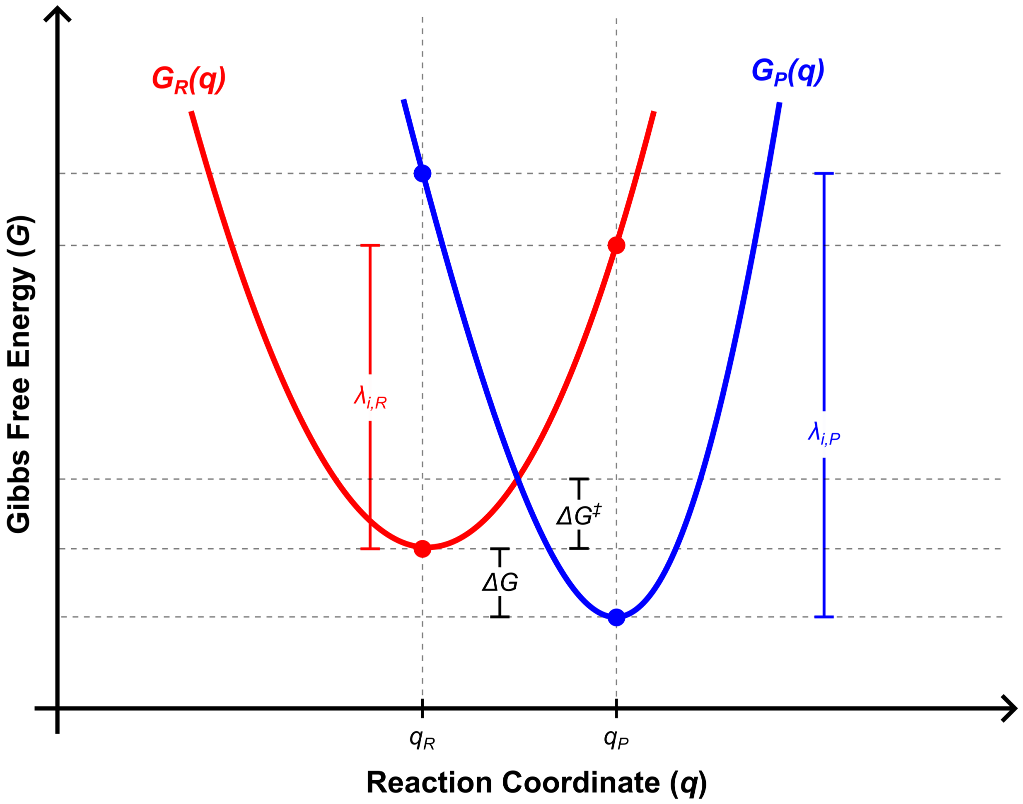

Fig. S1.: Marcus Theory energy diagram for outer-sphere electron transfer processes.

Based on Figure S1 and considering the following reaction in which the reactants R are A_{ox} and B_{red}, and the products P are A_{red} and B_{ox}:

$$A_{ox} + B_{red} \rightleftharpoons A_{red} + B_{ox} \tag{1}$$

The Marcus Theory expression for the energy barrier $\Delta G^{\ddagger}$ relative to the outer-sphere electron transfer from B_{red} to A_{ox} is:

$$\Delta G^{\ddagger} = \frac{(\Delta G + \lambda)^2}{4\lambda} \tag{2}$$

The reorganization energy λ can be divided into two contributions: the internal reorganization (λ_i) and external reorganization (λ_e). The former can be estimated by the following average value:

$$\lambda_i = \frac{\lambda_{i,\mathrm{R}} + \lambda_{i,\mathrm{P}}}{2} \tag{3}$$

where the reactants and products terms of internal reorganization are $\lambda_{i,\mathrm{R}}$ and $\lambda_{i,\mathrm{P}}$, respectively. Those are calculated from single-point electronic energies E according to:

$$\lambda_{i,\mathrm{R}} = [E_{\mathrm{A_{ox}}}(q_{\mathrm{A_{red}}}) + E_{\mathrm{B_{red}}}(q_{\mathrm{B_{ox}}})] - [E_{\mathrm{A_{ox}}}(q_{\mathrm{A_{ox}}}) + E_{\mathrm{B_{red}}}(q_{\mathrm{B_{red}}})] \tag{4}$$

$$\lambda_{i,\mathrm{P}} = [E_{\mathrm{A_{red}}}(q_{\mathrm{A_{ox}}}) + E_{\mathrm{B_{oxi}}}(q_{\mathrm{B_{red}}})] - [E_{\mathrm{A_{red}}}(q_{\mathrm{A_{red}}}) + E_{\mathrm{B_{ox}}}(q_{\mathrm{B_{ox}}})] \tag{5}$$

The external reorganization energy, which accounts the rearrangements in the solvent cavities, is calculate by:

$$\lambda_o = (332\ \mathrm{kcal\,\AA/mol})\left(\frac{1}{2r_{\mathrm{A_{ox}}}} + \frac{1}{2r_{\mathrm{B_{red}}}} - \frac{1}{r_{\mathrm{A_{ox}}} + r_{\mathrm{B_{red}}}}\right)\left(\frac{1}{D_{\mathrm{optical}}} - \frac{1}{D_{\mathrm{static}}}\right) \tag{6}$$

For which $D_{optical}$ and D_{static} are respectively the squared values for the water refractive index (1.33) and dielectric constant (78.36); also, the solvent cavity radii are denoted by r. The constant value of 332 kcal Å/mol arises from the amount of electric charge transferred, after unit corrections.

[1] Batista, V. *et al.* Marcus Theory with Gaussian and ADF – a Tutorial. 2016.

[2] Solé-Daura, A., Maseras, F. Straightforward computational determination of energy-transfer kinetics through the application of the Marcus theory. *Chemical Science*, 15(34), 2024.

[3] Zhu, Q. *et al.* Deciphering the active species and reaction mechanism in water oxidation catalyzed by a copper complex with redox-active ligands. *Inorganic Chemistry Frontiers*, 11(8), 2024.

S3. Alternative Structures

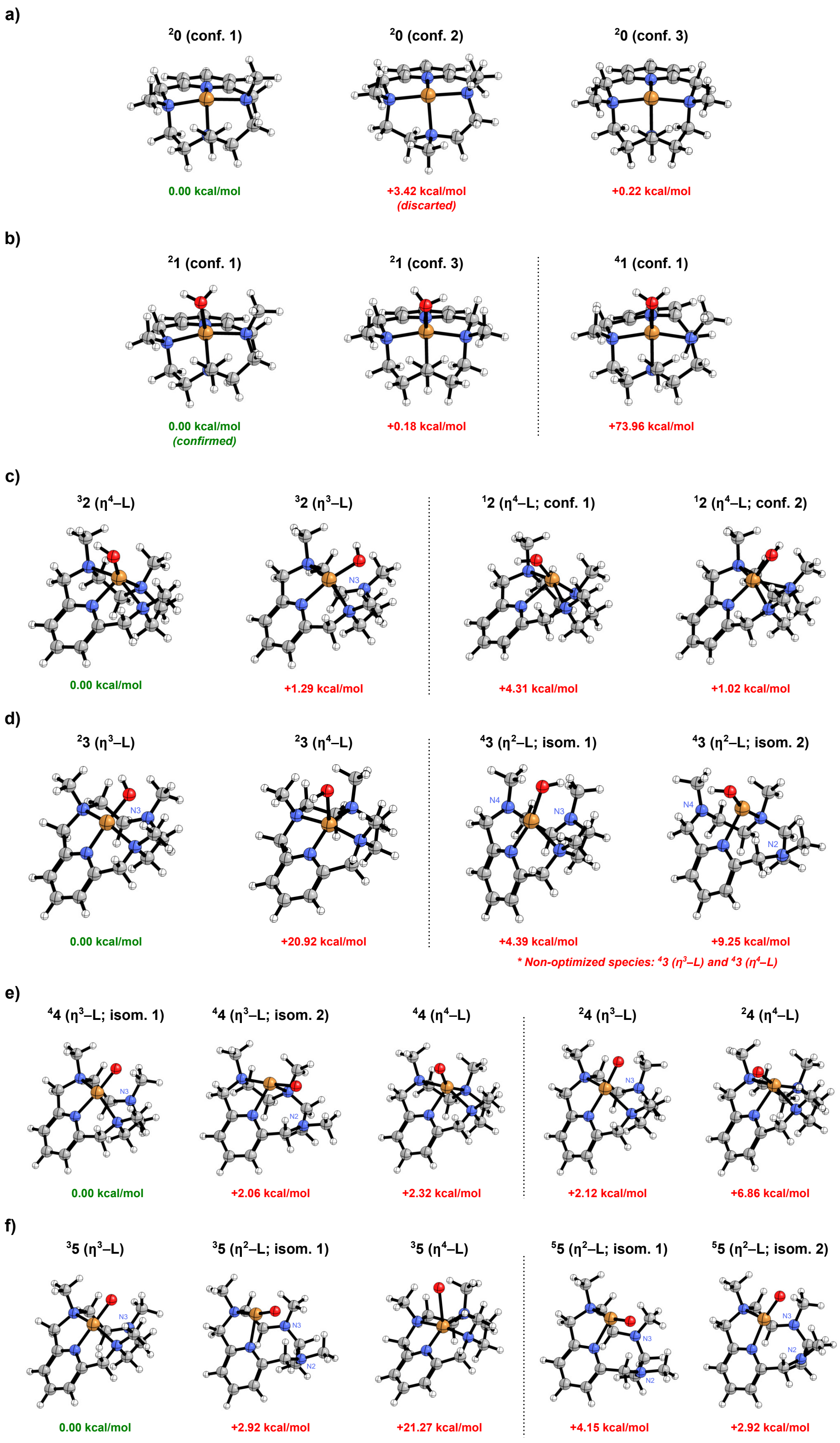

Fig. S2.: Alternative structures for the stationary points showed in the Figure 1.

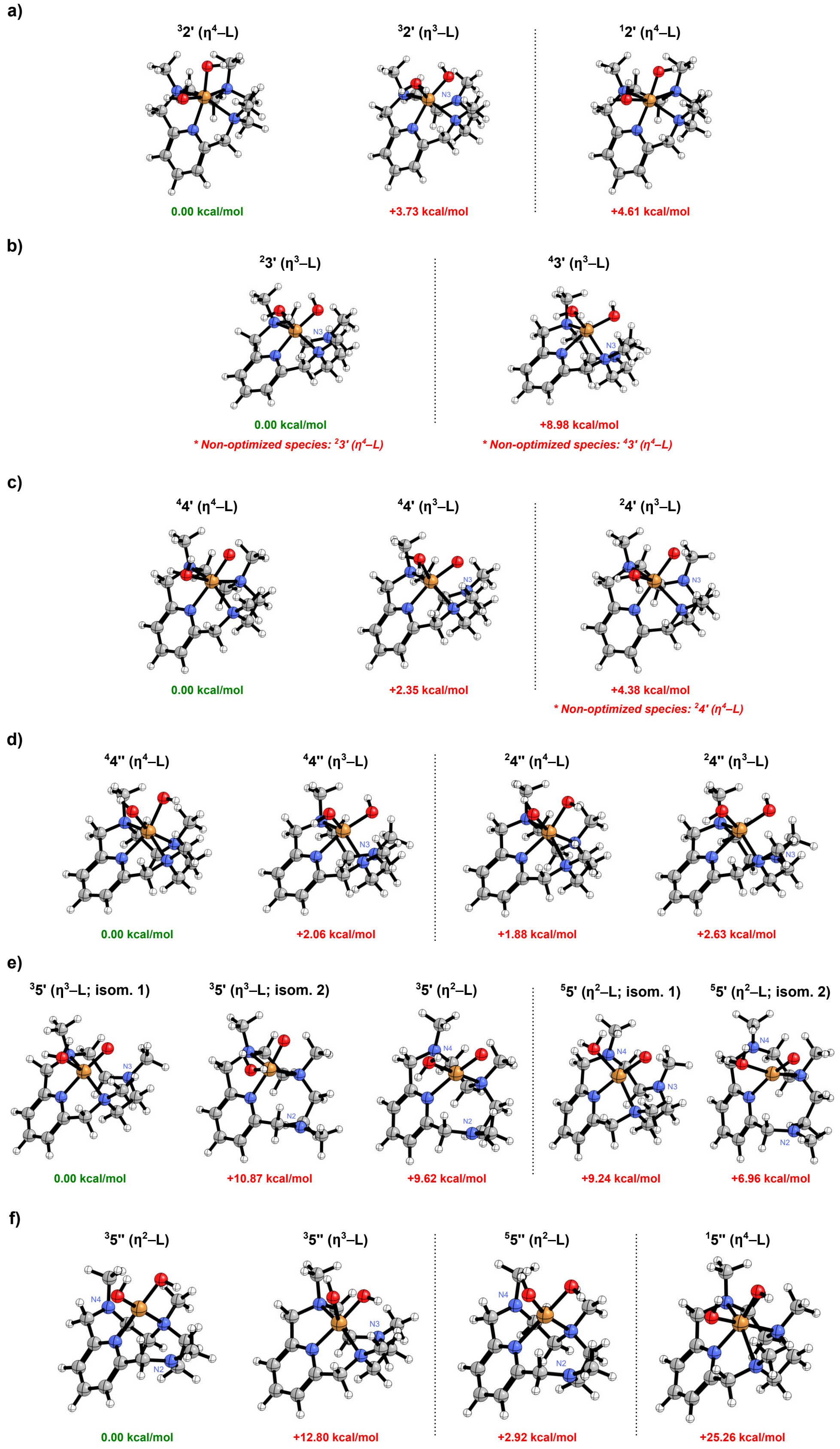

Fig. S3.: Alternative structures for the stationary points showed in the Figure 2.

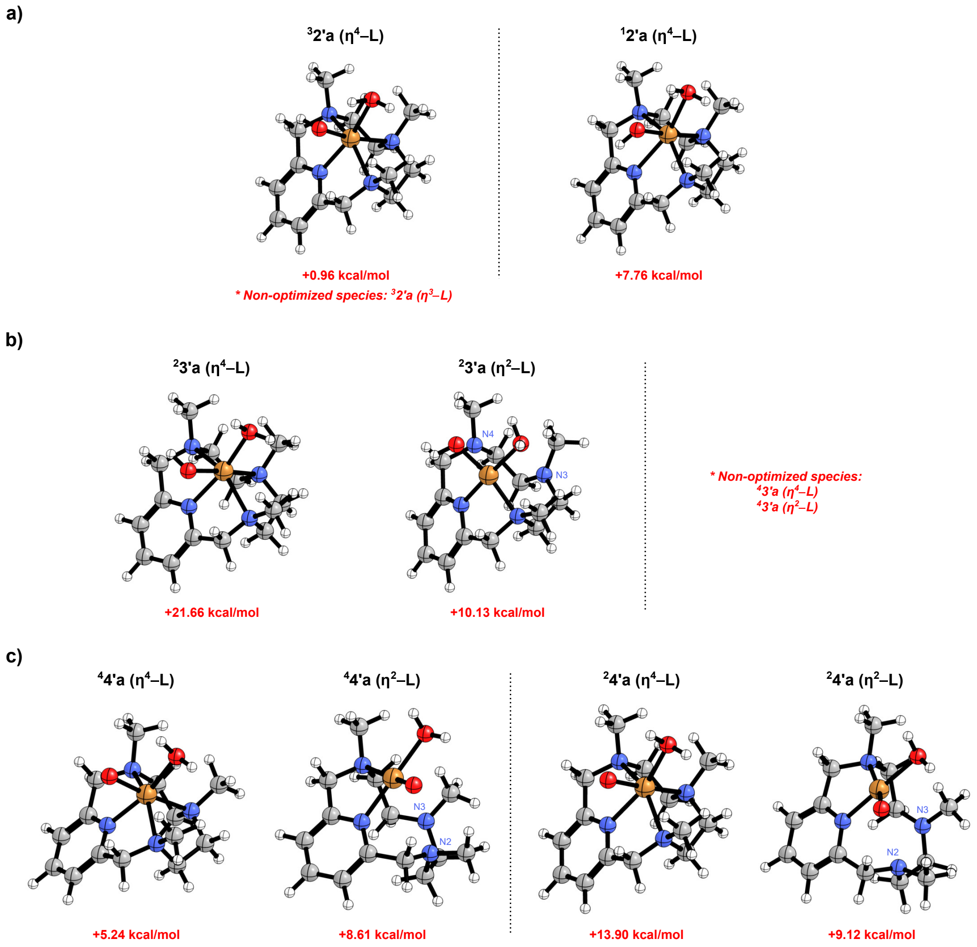

Fig. S4.: Alternative structures for the stationary points showed in the Figure 2, switching relative positions of the coordinated oxygens.

S4. Spin Densities

Table S2.: Calculated spin densities (M06L-D3/Def2-TZVP) of the investigated species from main text.

**Species**	Spin Densities				
	Cu	L	O1	O2	HPO_4
$^{2}\mathbf{1}$	0.60	0.40	0.00	—	—
$^{3}\mathbf{2}$	0.72	0.91	0.37	—	—
$^{2}\mathbf{3}$	0.00	0.95 (N3 = 0.80)	0.05	—	—
$^{4}\mathbf{4}$	0.60	1.24 (N3 = 0.73)	1.16	—	—
$^{3}\mathbf{5}$	−0.06	1.00 (N3 = 0.82)	1.06	—	—
$^{3}\mathbf{2}'$	0.69	0.98	0.25	0.08	—
$^{2}\mathbf{3}'$	0.00	0.92 (N3 = 0.79)	0.08	0.00	—
$^{4}\mathbf{4}'$	0.75	0.91	1.27	0.07	—
$^{4}\mathbf{4}''$	0.68	0.93	0.68	0.71	—
$^{3}\mathbf{5}'$	0.06	0.88 (N3 = 0.82)	1.06	0.00	—
$^{3}\mathbf{5}''$	0.00	2.00 (N2 = 0.80) (N4 = 0.80)	0.00	0.00	—
$^{3}\mathbf{RC}_5$	0.67	−0.62 (N2 = −0.79)	0.95	0.01	0.99
$^{3}\mathbf{TS1}_5$	0.63	−0.73 (N2 = −0.79)	0.65	0.43	1.02
$^{3}\mathbf{INT}_5$	0.56	0.74 (N2 = 0.78)	−0.14	−0.61	1.02
$^{3}\mathbf{TS2}_5$	0.00	0.97 (N2 = 0.80)	0.03	0.01	0.99

Table S2.: Calculated spin densities (M06L-D3/Def2-TZVP) of the investigated species from main text – continued.

**Species**	Spin Densities				
	Cu	L	O1	O2	HPO_4
^{3}**PC**$_5$	0.61	1.19 (N2 = 0.77)	0.16	0.03	0.01
^{3}**RC**$_{5'}$	0.61	−0.72 (N2 = −0.76)	1.12	0.09	0.90
^{3}**TS1**$_{5'}$	0.51	0.14 (N2 = 0.70) (N4 = −0.77)	1.14	0.13	0.08
^{3}**INT**$_{5'}$	−0.02	0.91 (N2 = 0.77)	1.07	0.00	0.04
^{3}**TS2**$_{5'}$	0.03	1.00 (N2 = 0.78)	0.76	0.19	0.02
^{3}**6a**	0.61	0.80 (N2 = 0.46)	0.45	0.14	—
^{3}**6**	0.54	0.46	0.67	0.33	—
^{3}**7**	0.48	0.16	0.65	0.71	—
^{4}**8**	0.56	1.40 (N3 = 0.80)	0.68	0.36	—
^{4}**9**	0.53	0.48	0.99	1.00	—

S5. Broken-Symmetry Calculations

In the following table, the results for broken-symmetry calculations are compiled. Notes: $E_{\mathrm{HS}} - E_{\mathrm{BS}}$ is the electronic energy difference between the high spin species and its broken-symmetry analogue. These values, along with the exchange coupling constant J_{AB}, are given in kcal/mol.

Table S3.: Broken-symmetry data for selected triplet species

Species	$E_{\mathrm{HS}} - E_{\mathrm{BS}}$	J_{AB}	Observations
$^{3}\mathbf{5}$	−0.329	0.328	Ferromagnetic coupling
$^{3}\mathbf{5}'$	−0.498	0.497	Ferromagnetic coupling
$^{3}\mathbf{5}''$	0.01	−0.01	Antiferromagnetic coupling
$^{3}\mathbf{RC}_{5}$	−4.94	4.61	Ferromagnetic coupling
$^{3}\mathbf{RC}_{5'}$	−4.20	4.17	Ferromagnetic coupling
$^{3}\mathbf{RC}_{5''}$	−2.77	2.61	Ferromagnetic coupling

S6. Additional Information on O–O Bond Formation

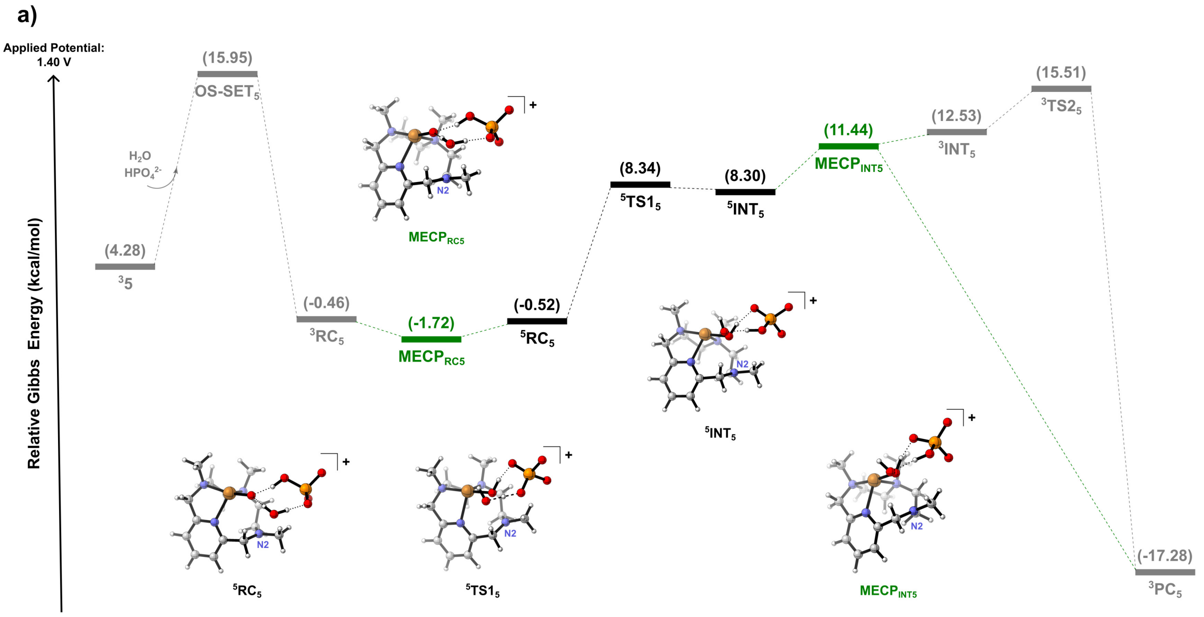

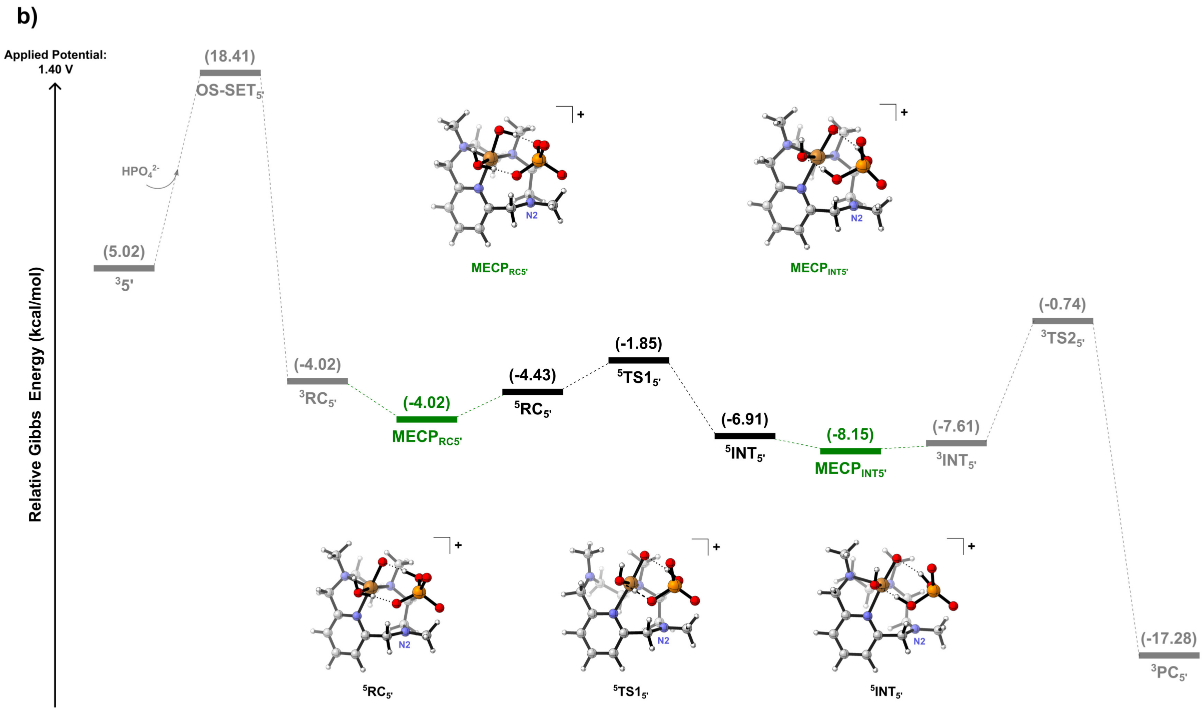

Fig. S5.: Gibbs free energy profiles of the Pathways ^{3}**5** (a) and ^{3}**5′** (b) – in grey – considering minimum energy crossing points with the quintet multiplicity surface – in black.

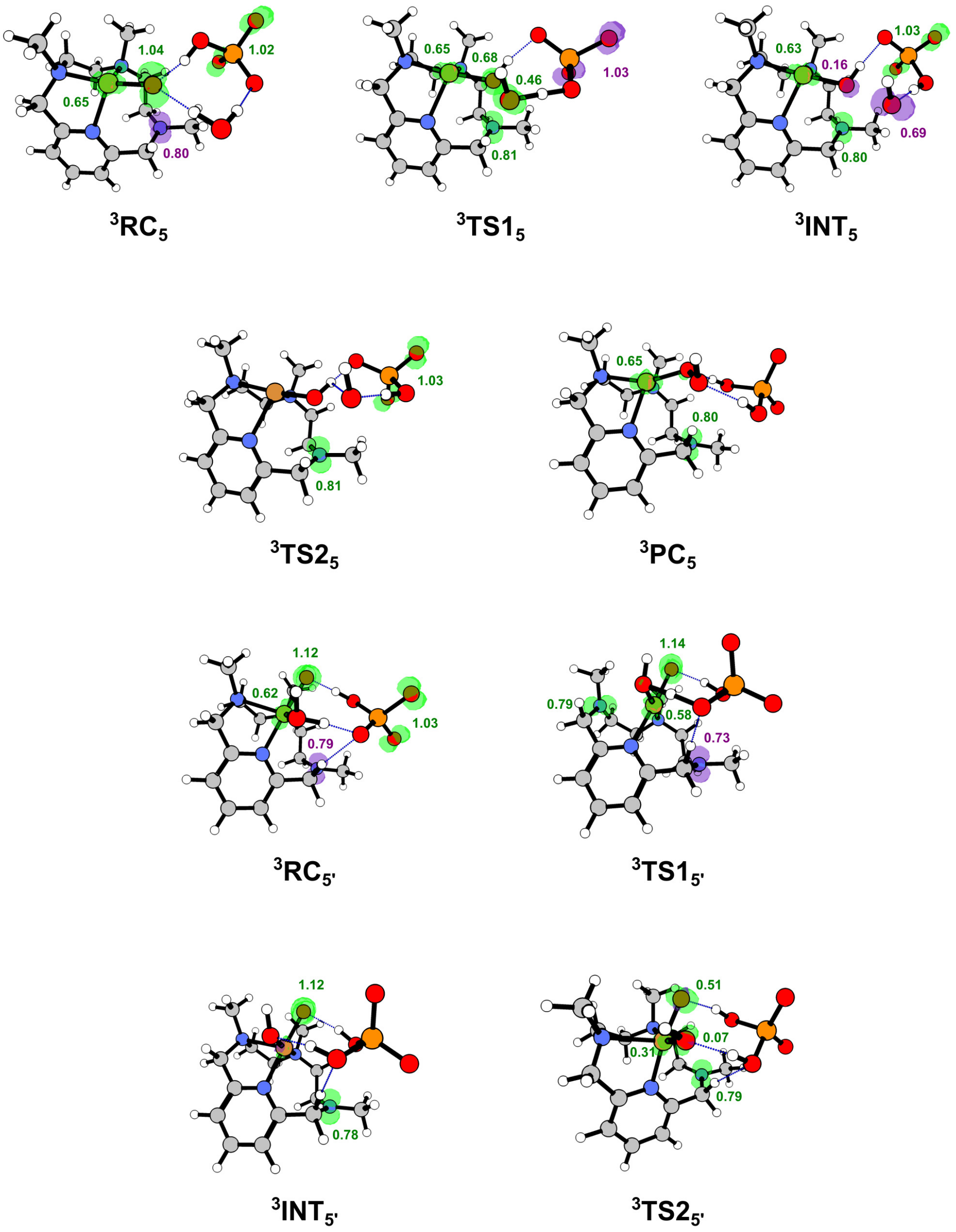

Fig. S6.: Spin density isosurfaces of key species of Figures 3 and 4 of the main text, obtained with B3LYP-D3/Def2-SVP level of theory.

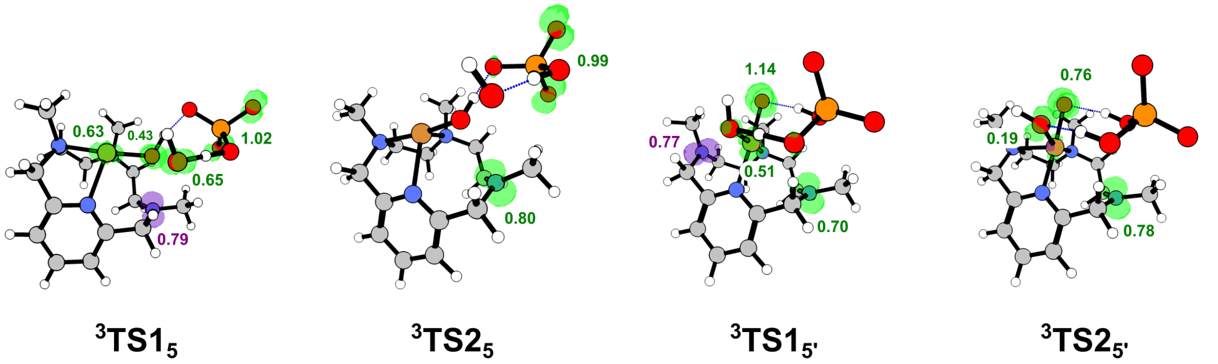

Fig. S7.: Spin density isosurfaces of transition states indicated in Figures 3 and 4 of the main text (obtained with M06L-D3/Def2-TZVP level of theory).

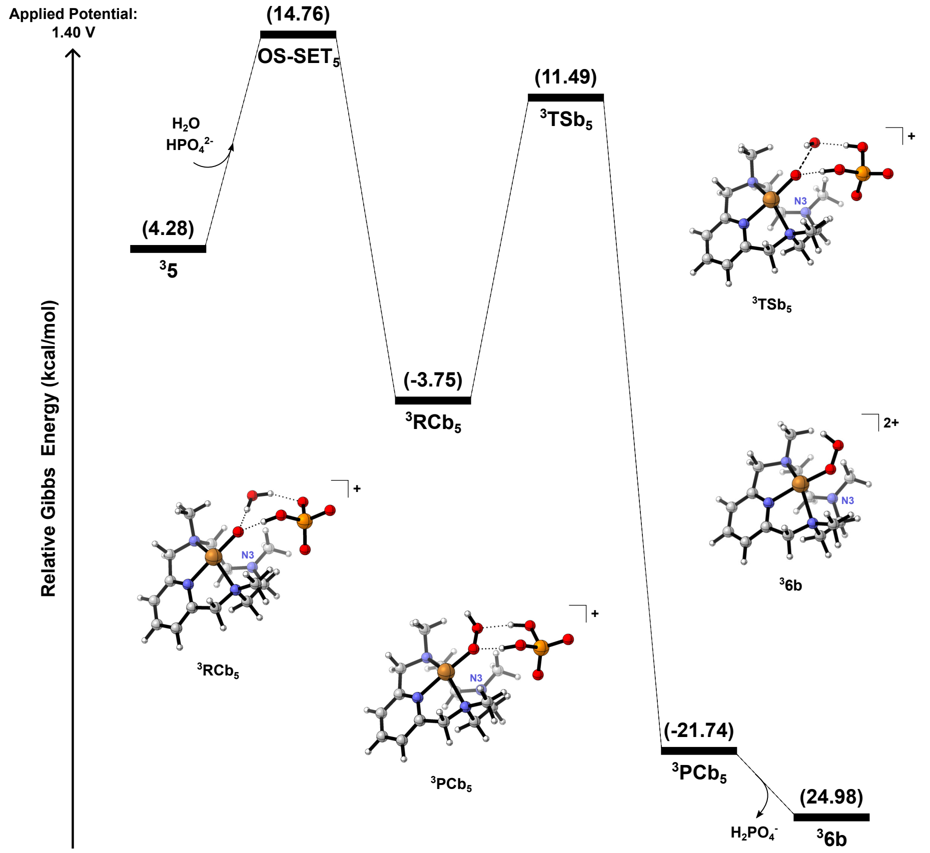

Fig. S8.: Gibbs free energy profiles of the alternative Pathway ^{3}**5**, in which the uncoordinated nitrogen is N3 instead of N2; the IRC of ^{3}**TSb**$_5$ was not conclusive, although the energy barrier is consistent with experimentally measured TOF.

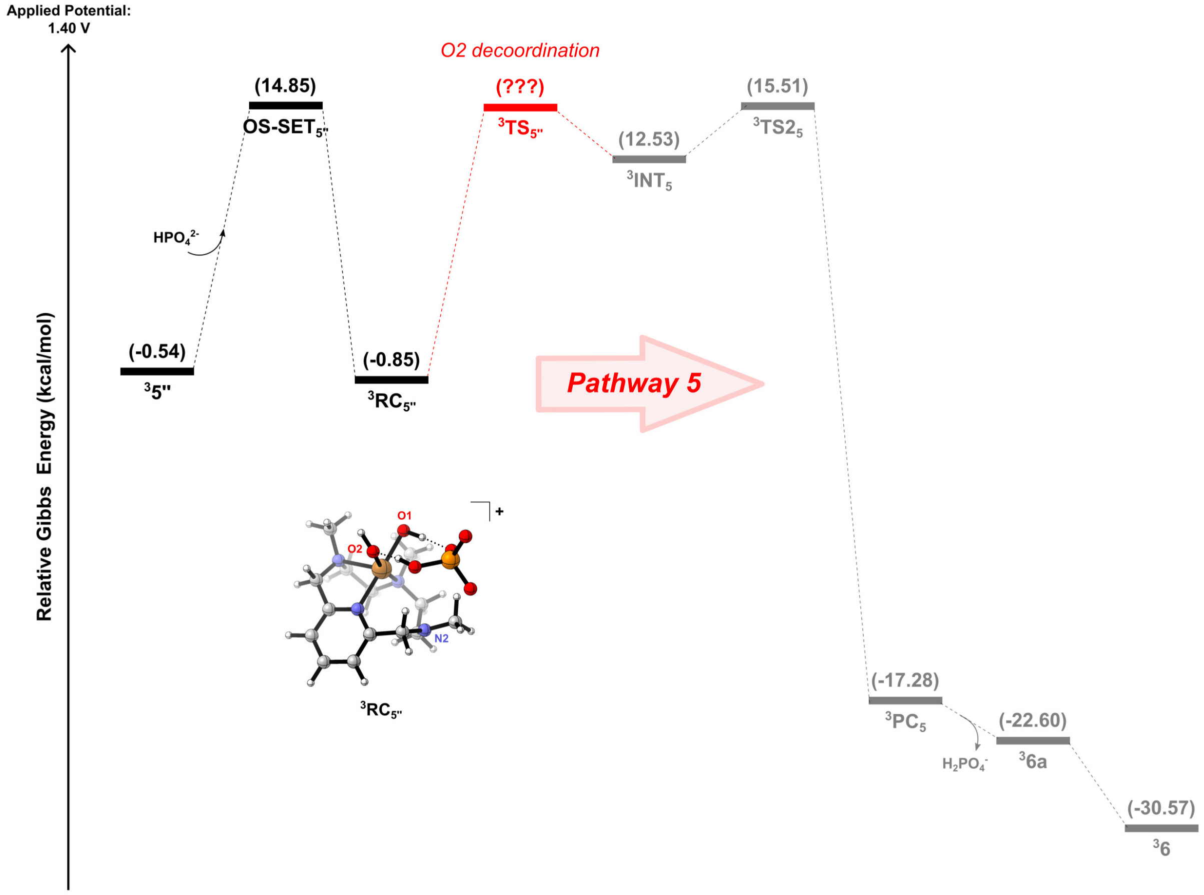

Fig. S9.: Gibbs free energy profile of the Pathway ^{3}**5**″. Regarding O−O bond formation, there was no new TS found; however, upon O2 dissociation from the complex (whose TS also wasn't found), the catalysis may proceed through Pathway ^{3}**5** – in grey color.

Table S4.: Charge transfer analysis of selected intermediates and TSs employing Hirshfeld charge (q_{H}) calculations.

**Species**	Atom	q_{H}
^{3}**INT**$_5$	Cu	0.667
	O1	-0.328
	O2	-0.234
^{3}**TS2**$_5$	Cu	0.606
	O1	-0.245
	O2	-0.289
^{3}**PC**$_5$	Cu	0.647
	O1	-0.220
	O2	-0.106

S7. Additional Information on 12-TMC Comparison

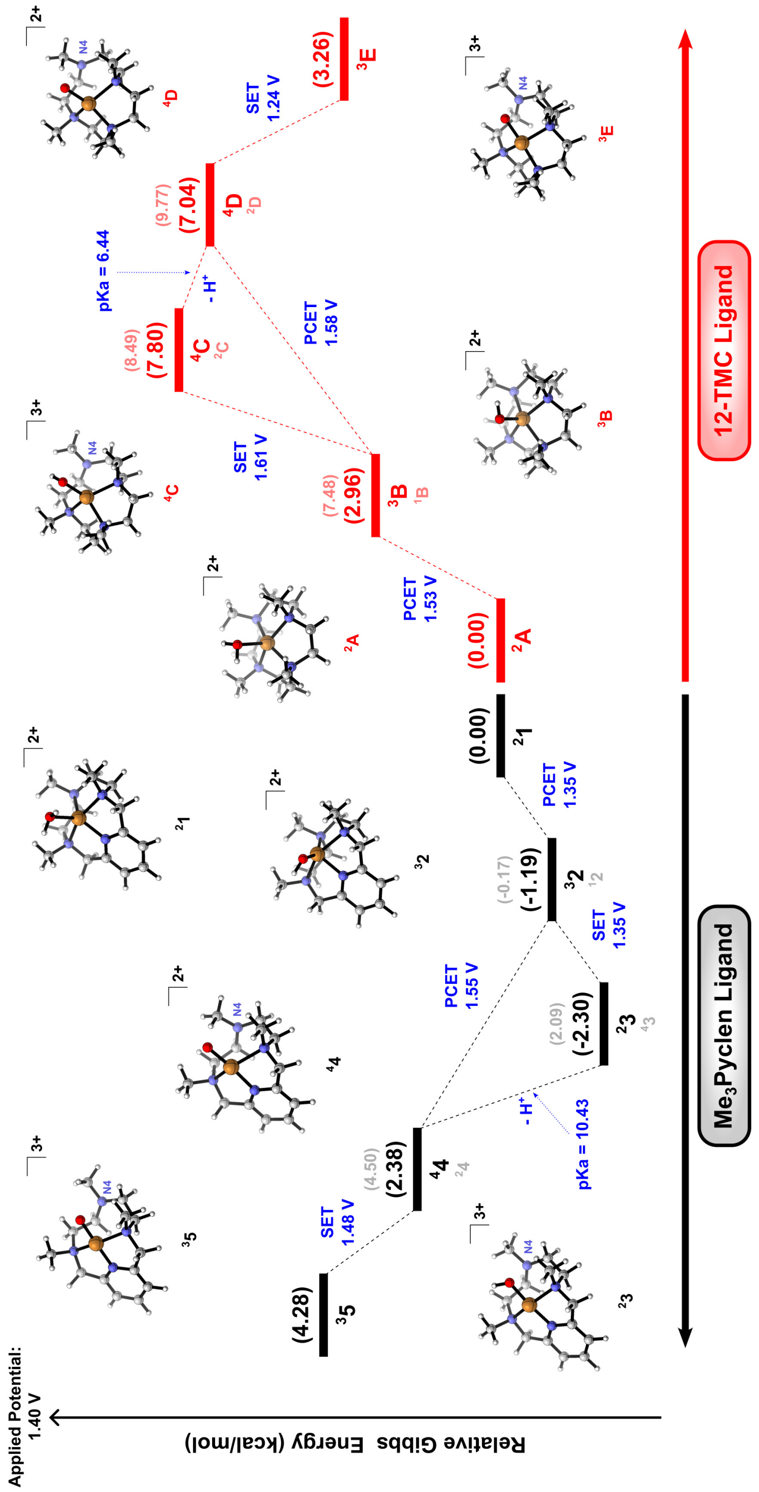

Fig. S10.: Gibbs free energy profiles of electrochemical activation for WO with different catalysts: 12-TMC (in red) *vs.* Me_3Pyclen (in black). Both were calculated at the same DFT level of theory.

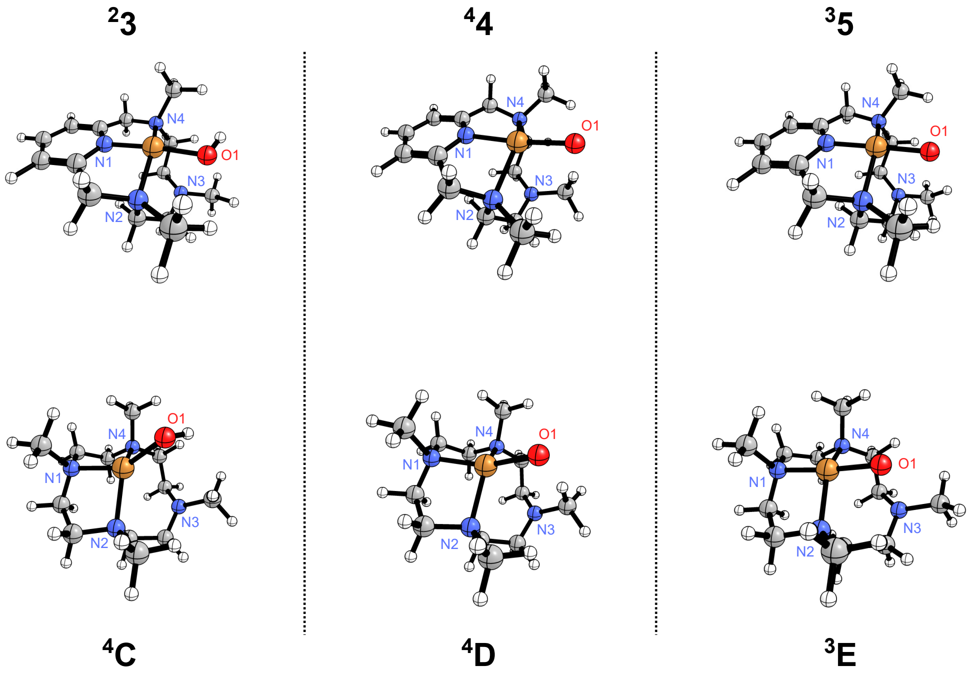

Fig. S11.: Optimized structures of selected η^3 intermediates for 12-TMC *vs.* Me_3Pyclen comparison.

Table S5.: Geometric comparison between selected η^3 intermediates of 12-TMC and Me_3Pyclen catalysis, based on Figure S10.

**Species**	Angle	Value
^{2}**3**	N1-Cu-O1	174.94°
	N2-Cu-N4	153.00°
^{4}**C**	N1-Cu-O1	145.64°
	N2-Cu-N4	122.04°
^{4}**4**	N1-Cu-O1	173.12°
	N2-Cu-N4	137.33°
^{4}**D**	N1-Cu-O1	156.34°
	N2-Cu-N4	126.73°
^{3}**5**	N1-Cu-O1	178.80°
	N2-Cu-N4	151.13°
^{3}**E**	N1-Cu-O1	173.73°
	N2-Cu-N4	140.46°
